# The Optical and Spectroscopic Properties of Fuchsite, Spodumene, and Lepidolite from Northern Scandinavia (Kautokeino, Kaustinen, Kolmozero)

**DOI:** 10.3390/ma16144894

**Published:** 2023-07-08

**Authors:** Miłosz Huber, Daniel M. Kamiński, Urszula Maciołek

**Affiliations:** 1 Department of Geology, Soil Science and Geoinformacy, Faculty of Earth Science and Spatial Management, Maria Curie-Skłodowska University, 2d/107 Kraśnickie Rd., 20-718 Lublin, Poland; 2 Faculty of Chemistry, Maria Curie-Skłodowska University, 2/508 Maria Curie-Skłodowska Sq., 20-031 Lublin, Poland; daniel.kaminski@mail.umcs.pl; 3 Analytical Laboratory, Institute of Chemical Sciences, Faculty of Chemistry, Maria Curie-Skłodowska University, 3/27 Maria Curie-Skłodowska Sq., 20-031 Lublin, Poland; urszula.maciolek@mail.umcs.pl

**Keywords:** LCT pegmatites, Kautokeino, Kaustinen, Kolmozero, N Scandinavia, spodumene, fuchsite, lepidolite, crystal structure, optical properties

## Abstract

Li-Ce-Ta (LCT) pegmatites containing lithium mineralization in the form of spodumene and lepidolite, as well as fuchsite, from the regions of northern Scandinavia (N Norway, N Finland, N Russia) were studied. Detailed analyses of the chemical compositions of these minerals were carried out, involving scanning electron microscopy (SEM) with energy-dispersive spectroscopy (EDS), Fourier transform infrared (FTIR) spectroscopy with attenuated total reflectance (ATR), and X-ray photoelectron spectroscopy (XPS) studies. Their crystal structures were confirmed with the X-ray diffraction technique. Studies involving microscopy were also carried out, indicating the optical features of these minerals. Based on the analyses carried out in the studied rocks, the characteristics of these minerals were determined, as well as the crystallization conditions. This research indicates that the N Scandinavian area is prospective and may lead to further discoveries of this type of pegmatite in the studied region.

## 1. Introduction

In northern Scandinavia, pegmatite veins containing rare lithium and schists with chromium minerals are found in the regions of northern Norway [1], Finland [2], and the Kola Peninsula in Russia [3], among rocks constituting the cratonic basement of the Northern Fennoscandia [4]. These rocks contain minerals such as mica (lepidolite, fuchsite, muscovite) and pyroxene (spodumene), which co-occur with quartz, plagioclase, orthoclase, and several ore minerals: oxides (e.g., columbite, tantalite, cassiterite, sillenite, clarkeite) and sulfides (pyrite, chalcopyrite). Their thickness reaches several meters, and they cut through their metamorphic host rocks [5]. The previous study of these rocks was mainly concerned with analyses for the needs of economic geology [6]. The purpose of this article is a detailed analysis of the mineralogy of these rocks and a discussion of the crystalline structure characteristics of spodumene, lepidolite, and fuchsite.

## 2. Study Area and Massifs Geology

**Figure 1:**
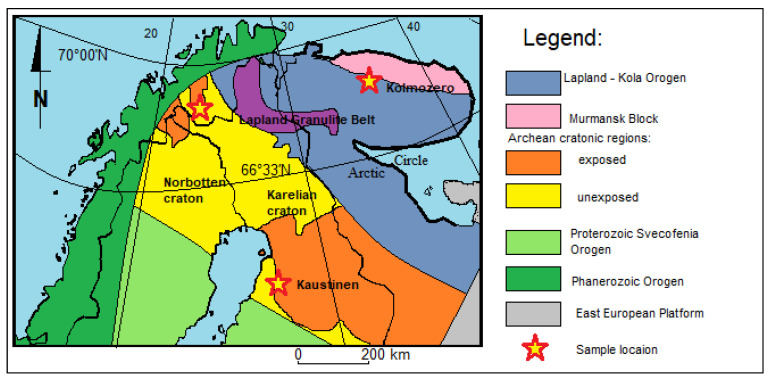
Simplified geological–tectonic map of N Scandinavia (after [37]) with the sample locations.

The northern part of the Scandinavian Peninsula is part of the Fennoscandia (Figure 1). In this region, Archean and Proterozoic rocks are exposed, forming various types of gneisses that constitute the basement of the Baltic Shield [3,7,8,9,10,11,12,13,14]. In the Fennoscandia, numerous intrusions have been found, such as the Tonalite Trondheimite Granodiorites of Murmansk (3.75 Ga) [3] and Jergul (2.97 Ga) [15], Patchenvarek anorthosites (2.92 Ga) [16], and enderbites (2.83 Ga) [9], with layered Paleoproterozoic intrusions in Monchegorsk (2.50 Ga) [17]. These rocks metamorphosed in the amphibolite and granulite facies [18,19]. Alkaline metavolcanic rocks and granitoid intrusions occur in the vicinity of the discussed pegmatite sites [20,21,22]. This is the case in the Kaustinen and Kolmozero areas, where numerous pegmatite veins can be found in these rocks [23,24,25,26]. In the Kaustinen area (Finland) and the Kolmozero area, there are pegmatite veins with lithium–cesium–tantalum mineralization (LCT), which are up to several meters thick and 100 m or more in length. In the Kautokeino area, schists containing chromic mica are visible. The age of Kautokeino and Kaustinen rocks spans the range of 1.9–1.8 Ga [27,28,29]. These rocks are exposed on the surface or covered by Pleistocene sediments [30,31]. In these locations, high-relief landscapes have developed [32,33,34,35,36].

## 3. Materials and Methods

Rock samples were collected between 2018 and 2022. During this period, the discussed massifs were visited, and the geological documentation of the samples was also carried out. In Scandinavia, rock samples were taken from the site of their occurrence. The selected rocks were targeted for thin-section preparations. Subsequently, the minerals were subjected to analyses using a Leica DM2500P (Heerbrugg, Switzerland) polarizing optical microscope [38,39,40,41] and with a scanning electron microscope, a Hitachi SU6600 (Tokyo, Japan), equipped with energy-dispersive spectroscopy (EDS) [42,43]. The samples were analyzed under a low vacuum (10 Pa), 15 kV accelerating voltage, and beam diameter of 0.2 µm. A total of 525 analyses were performed using the microprobe (at Kautokeino, 72; at Kaustinen, 179; and at Kolmozero, 274). The selected minerals were separated and analyzed with single-crystal X-ray diffraction. Data were collected using a Rigaku diffractometer (Tokyo, Japan) [44] with CuKα radiation (λ = 1.54184 Å) at 293 K. Crystallographic refinement and data collection, as well as data reduction and analysis, were performed with a CrysAlisPro v42 [45]. The selected single crystals were mounted on the nylon loop with oil. The structures were determined by applying direct methods using the SHELXS-86 program and refined with SHELXL [46,47] in Olex2 software [48]. Table 1 provides the experimental details for the single crystals’ X-ray measurements. These samples were also examined with the Fourier transform infrared (FTIR) technique [49,50]. The samples were measured using a Nicolet 8700A Thermo Scientific Shimadzu FTIR spectrophotometer (Waltham, MA, USA) equipped with an attenuated total reflectance (ATR) accessory, a Smart Orbit™ diamond ATR QATR-S (Riyadh, Saudi Arabia) (wideband diamond crystal). Every spectrum was obtained from 256 records at a 4 cm^−1^ resolution ranging from 4000 to 400 cm^−1^. The elaboration of a spectrum was carried out using the OMNIC program. Optical and microscopic studies were performed at the Department of Geology, Soil Science, and Geoinformation of the Institute of Earth and Environmental Sciences, and crystal chemistry studies were performed at the Department of Crystallochemistry, Faculty of Chemistry, Maria Curie-Skłodowska University, in Lublin. The UHV multi-chamber system (Prevac, Poland) was used to carry out the X-ray photoelectron spectroscopy (XPS) measurements. Molybdenum mounts were used to hold the measured samples. The chamber pressure was 5 × 10^−9^ mbar during analysis. The mineral surface was excited with X-rays (Al Kα, 1486.6 eV) from a Scienta SAX-100 X-ray source equipped with an XM 650 X-ray monochromator. The hemispherical electron analyzer R 4000 (Scienta, Uppsala, Sweden) operating in sweep mode was used to detect photons arriving from the sample. The energy of the spectra was calibrated using the C1s aliphatic carbon peak, EB = 285 eV. The CasaXPS v2.3.23-PR1 software from Casa Software Ltd. (London, UK) was used to analyze the measured data. The full width at half maximum (FWHM) and relative peak shift were fixed in the fitting process [51,52].

## 4. Results

### 4.1. Host Rock Petrology

The examined lithologies are located in the Kolmozero–Voronya Greenstone Belt, Pohjanmaa Schist Belt (Kaustinen Pegmatites), and Kautokeino Greenstone Belt. The LCT pegmatites from the Kolmozero area are located in the Kolmozero–Voronya Greenstone Belt, separating the Murmansk block from the Kola block, composed of mixed metasediments and metavolcanites. These formations are intersected by numerous intrusions of alkaline and acidic rocks. The discussed LCT pegmatites intersect the alkaline rocks of the Patchemvarek anorthosite intrusion. This intrusion is located on the border of the Kolmozero block with the plagiogranites of the Murmansk block [53]. These rocks are in direct contact with biotite gneisses and amphibolites of the Kolmozero–Voronya Belt. There are fine-grained tourmaline-muscovite granites with pegmatite apophyses rich in tourmaline, garnet, and apatite. The studies of Kudrashov et al. [54] indicated that these are pegmatites of hydrothermal–metasomatic origin [55,56].

The Kaustinen pegmatite province is located in Western Finland. It is situated among supracrustal rocks belonging to the Pohjanmaa Schist Belt [57,58]. It is surrounded by the Vaasa granitoid complex to the west and Central Finland granitoides to the east [6]. The Pohjanmaa Schist Belt is composed of micaceous schists and gneisses, along with metavolcanic rocks. These rocks were metamorphosed under the amphibolite facies of 1.89–1.88 Ga [59]. The lithium-rich pegmatites from the Kaustinen province belong to the albite spodumene type according to the classification of Černy and Ercit [55]. They were formed during the metamorphism of the rocks of the Pohjanmaa belt. The pegmatites form a complex of veins cutting the rocks of the Pohjanmaa Käpyaho belt and others [60,61,62,63].

In the region of the Palaeoproterozoic Kautokeino Greenstone Belt, where muscovite-fuchsite and quartz-orthoclase schists are located between the gneissic Ráiseatnu Complex (1868–1828 Ma) to the west and the metaplutonic Jergul Complex to the east (tonalite–trondhjemite–granodiorite–granite plutonic rocks formed between 2975 and 2776 Ma). In the Kautokeino Greenstone Belt, metasedimentary–metavolcanic rocks are present, with numerous mafic intrusions [27]. The Masi Formation in the Archean basement is formed of a quartz-feldspar conglomerate with muscovite interbedding and ore mineralization composed of iron and copper sulfides (pyrite, chalcopyrite). It is intersected by the mafic sills of the Haaskalehto formation (2220 Ma age). [64] Among these formations are the discussed fuchsite-rich rocks, which may have formed as an alteration product of detrital chromite grains [65].

#### 4.1.1. Kautokeino

**Figure 2:**
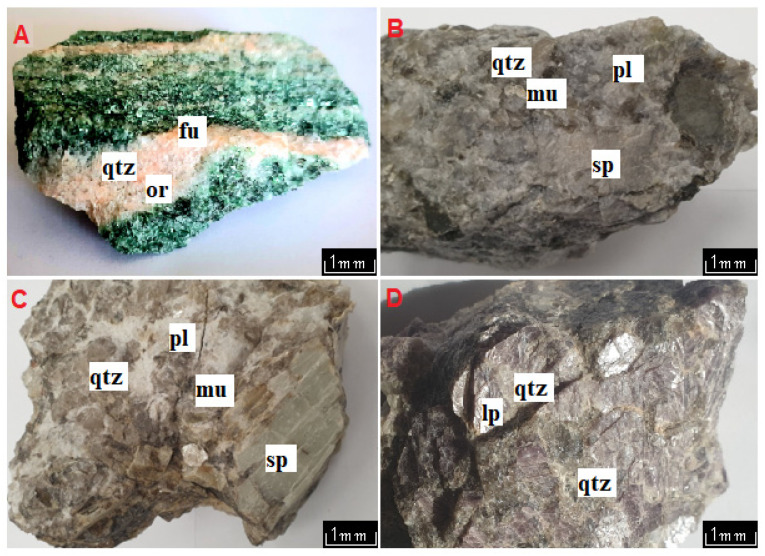
Macroscopic images of rocks under investigation. Schist with fuchsite from Kautokeino (**A**), pegmatite with lepidolite from Kaustinen (**B**), and pegmatite from Kolmozero with spodumene (**C**) and lepidolite (**D**). Abbreviations: fu—fuchsite, sp—spodumene, lp—lepidolite, qtz—quartz, mu—muscovie, pl—plagioclase, or—orthoclase.

The paleoproterozoic metamorphic formations classified as the Alta-Kautokeino Greenstone Belt are exposed in the Kautokeino region. Among these formations, there are mica schists [66] containing fuchsite (Figure 2A). This rock is an intense green-pink, characterized by layering resulting from a gneissic, streaky texture. The rock has a grano-lepidoblastic texture and is also characterized by a glomeroblastic, locally diablastic texture. Under an optical microscope, large quartz crystals are visible, forming irregular, hooked aggregates in contact with each other. Opaque minerals, such as pyrite and chalcopyrite, are visible between the muscovite and fuchsite. Close to the quartz crystals, microcline and Na-rich plagioclase are also present, forming leucocratic zones. In the interstices of plagioclase and microcline, small crystals of epidote are encountered. In addition to these zones are areas richer in femic minerals. They are represented by aggregates of biotite, which co-occur with fuchsite to form a streaky structure in the rock. These minerals form aggregates, resulting in interlaced streaks in the discussed rock. Opaque minerals can be observed, and zircon and apatite are woven into the biotite flakes. The detailed results of the phase studies in the micro-area are discussed below.

#### 4.1.2. Kaustinen

In the Kaustinen area, pegmatites with a silicon-gray color, coarse crystalline structure, and compact, disordered texture are exposed among the gneisses. Their age was determined to be 1.79 Ga (U-Pb method) [67]. Under an optical microscope, the quartz crystals form large grains in contact with each other and closely interlocking. The quartz in the studied rock has wavy extinction. Alongside these are large orthoclase crystals adjacent to the quartz (Figure 2B). They are accompanied by much smaller tabular grains of Na-rich plagioclase, often between the orthoclase and quartz. Femic minerals are represented by muscovite forming large clusters of flakes, in the background of which fine zircon crystals are visible. Muscovite crystals are near the pyroxene. Biotite is also visible in the form of small flakes, usually between large pyroxene crystals. The pyroxenes in the discussed rock form large crystals, reaching several centimeters in size. They are represented by spodumene (and hypersthene). The spodumene forms compact crystals with jagged boundaries in which quartz and muscovite are visible. The hypersthene is anhedral. Opaque minerals (columbite-tantalite, sillenite) are visible in the form of small crystals close to the quartz, sometimes also forming solid inclusions, accompanying zircon. The detailed results of the micro-area phase studies are discussed below.

#### 4.1.3. Kolmozero

In the area of the Kola Peninsula between the Kola and Murmansk blocks is the Archean Kolmozero–Voronya Greenstone Belt. Adjacent to the LCT pegmatites are Archean gabbro-anorthosites and granitic rocks. The analyzed pegmatites are dated to 1.90–1.86 Ga [68]. The pegmatites containing spodumene and lepidolite are cream-gray-colored rocks with a coarse crystalline texture and a compact, disorderly texture (Figure 2C). In thin sections, there are large crystals of quartz interlocking with each other. The quartz forms clumped aggregates in the space between the other phases. Alongside these minerals are visible crystals of Na-rich plagioclase. In the described rock, orthoclase, usually reaching a considerable size, is also visible near the other leucocratic minerals. Among the potassium feldspars, microcline usually forms small crystals. Alongside these minerals, flakes of biotite and muscovite can be identified, interspersed among the quartz and plagioclase. Biotite is much less abundant than muscovite, which predominates. In the pegmatites with lepidolite, this mineral accompanies muscovite, forming deformed flake aggregates of varying sizes. Large crystals of spodumene, reaching several centimeters in length, are visible alongside these minerals. They are surrounded by biotite, lepidolite, and plagioclase. In addition, small crystals of opaque minerals are visible near muscovite flakes occurring in interstices of quartz and feldspar. Zircon crystals are also visible within mica flakes. Accessory apatite forms small crystals co-occurring with femic minerals.

### 4.2. SEM-EDS Analyses

**Figure 3:**
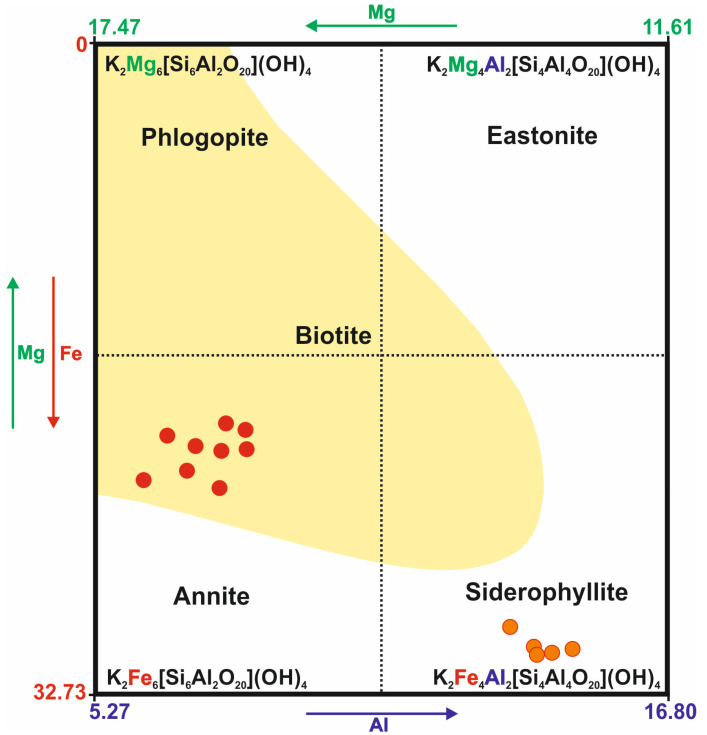
Mineral composition of trioctahedral micas from Kautokeino schists. The element content is expressed in wt.%. The yellow area represents the composition of the most frequent, naturally occurring biotites [71].

In the case of the Kautokeino rocks, the examined trioctahedral mica can mainly be classified as annite and siderophyllite [69,70,71] (Figure 3). These micas co-occur with fuchsite-forming overgrowths (Table A1).

**Figure 4:**
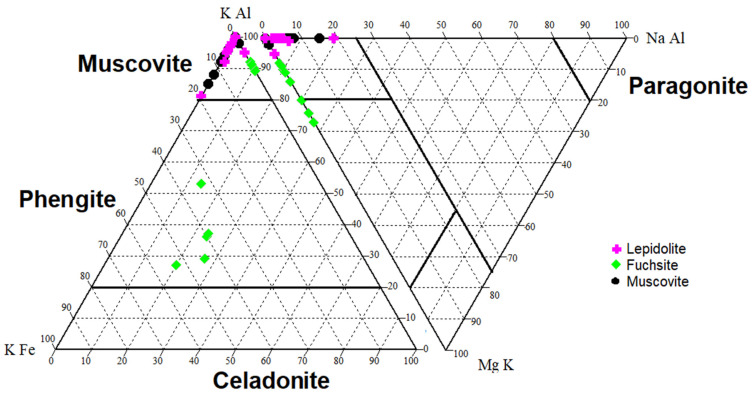
Type of dioctahedral micas examined in the discussed rocks (based on [72,73], modified by the authors). The element content is expressed in wt.%.

Dioctahedral micas are represented by muscovite with fuchsite and lepidolite (Table A1). Muscovite usually shows a low Na^+^ content (up to 5 wt.%). In the rocks from Kautokeino, in addition to muscovite, it was found that all the chromic micas examined were fuchsites. Some of the examined chromium micas have a composition characteristic of paragonite (Figure 4). In addition, an admixture of clinozoisite and epidote (mainly in the Kaustinen pegmatites, Table A1) was found in the discussed rocks.

The examined pyroxenes are mainly represented by spodumene, with an Na content of up to 5 wt.%. In the examined samples, spodumene dominates, while admixtures of jade particles occur in small amounts (Table A2). Hypersthene was also found in the latter.

The accompanying leucocratic minerals are represented by quartz, usually with an admixture of up to 4% of aluminum oxide. The plagioclase is represented by albite with a small admixture of oligoclase (4%), as well as andesine (6%) and labradorite (6%). The labradorite is an admixture found mainly in the pegmatites from Kaustinen. Accessory minerals are represented by zircon and apatite. Zircon crystals are mainly found in close association with muscovite flakes. Phosphates are represented mainly by a variety of hydroxyapatites, with approximately 3% carboxy apatite and 2% fluoroapatite.

In addition, opaque minerals were found in the exanimated rocks. In the Kolmozero pegmatite, columbite ((Fe,Mn)Nb_2_O_6_) forms euhedral crystals, usually close to spodumene, quartz, and plagioclase [2]. Along with columbite, tantalite is visible. Tantalite ((Fe, Mn)Ta_2_O_6_) is replaced with bixbyite in the oxidation zone. Magnetite was also found. The sample also presents clarkeite (Na,Ca,Pb)_2_(UO2)_2_(O,OH)_3_ in the vicinity of apatite and sillenite Bi_12_SiO in the vicinity of femic minerals.

The opaque minerals in the investigated rocks are represented by multiple phases. In the case of the schists from Kautokeino, the opaque phases include cassiterite and pyrite. In the pegmatite from Kaustinen, the opaque phases include magnetite and ilmenite, accompanied by titanite. Trace or minor cassiterite was also found, as well as galena, sphalerite, and chalcopyrite, which, together with barite, form disseminations. Columbite and tantalite were also found, although in smaller amounts relative to the Kolmozero pegmatite.

### 4.3. Optical Properties of the Discussed Minerals

**Figure 5:**
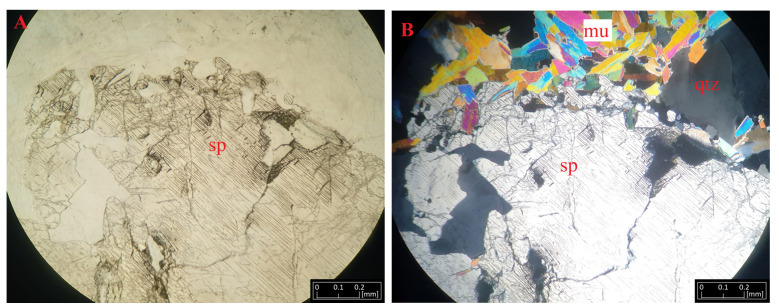
Microphotographs of spodumene (sp) from Kaustinen under transmitted light (**A**) and polarized light (**B**) accompanying quartz (qtz) and muscovite (mu) crystals.

Spodumene forms large xenomorphic crystals, with sizes reaching several cm. This mineral usually has a light green color. Sometimes, it resembles plagioclase, which, when undergoing sericitization, also has a slight gray-green tint. In the microscopic image, it sometimes forms a diablastic texture according to the (100) miller index. It is usually found near micas represented by muscovite, lepidolite, and, less often, biotite (Figure 5). The schist is highly visible. Small admixtures of Fe-oxides can be seen along the schistosity of the rock. The straw color on the thin section has a clear, positive relief, with darkening extinction.

**Figure 6:**
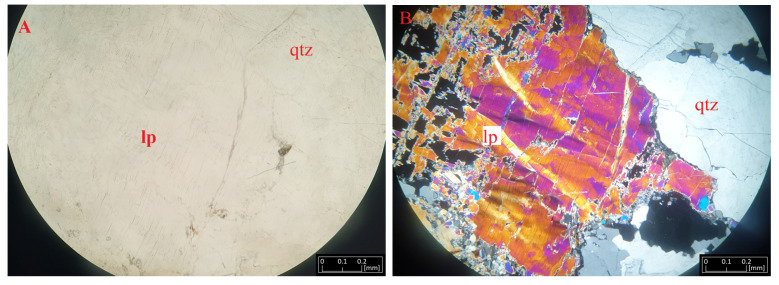
Microphotographs of lepidolite (lp) from Kolmozero under transmitted light (**A**) and polarized light (**B**) with quartz (qtz).

Lepidolite forms large lamellar aggregates, usually colored pearly pink. Macroscopically, this mineral forms flakes reaching up to 1 cm in size. It is usually quite visible in the rock due to its coloration and luster. In the microscopic image, it forms numerous adhesions of varying sizes. It is present with spodumene, near plagioclase and quartz. Between the mica flakes, rutile be observed. In the thin section, it is colorless, with a faint, negative relief. Under polarized light, it shows second-order interference colors, optically resembling biotite (Figure 6). Our microscopic observations of lepidolite showed some deformation of its lamellae due to dynamic processes.

**Figure 7:**
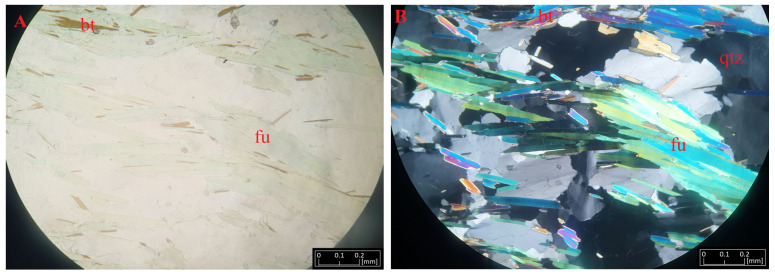
Microphotographs of fuchsite (fu) from Kautoteino under transmitted light (**A**) and polarized light (**B**) near biotite (bt) and quartz (qtz) crystals.

Fuchsite forms fine, scaly accumulations, the size of which reaches several millimeters. Macroscopically, it is colored green and has a pearly luster. It is highly visible against the background of biotite and feldspar in the investigated rocks. Fine zircon grains are visible in the background of the fuchsite aggregates (Figure 7). In the microscopic image, they form polysynthetic adhesions with muscovite occurring between quartz and orthoclase. The sample shows pleochroism with a delicate greenish (β)-bluish (α) coloration. Under polarized light, it shows intense second-order interference colors, making it similar to muscovite.

### 4.4. Spectroscopic Properties of the Minerals under Investigation

**Figure 8:**
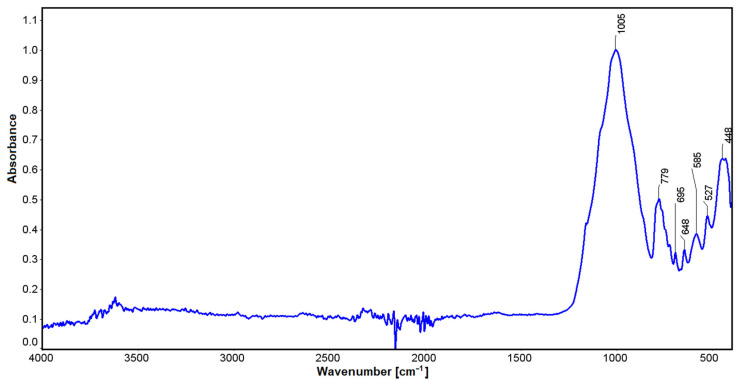
IR spectrum for the Kaustinen spodumene.

Infrared studies carried out for spodumene showed some small oscillations at a wavelength of 3612 cm^−1^, which can be explained by the influence of water. Values in the vicinity of 1005 cm^−1^ may be related to stretching vibrations for silicon-oxygen tetrahedra [74]. Vibrations in the vicinity of 779 cm^−1^ can be correlated with Si-O stretching vibrations. Similarly, for lengths of 647 cm^−1^ to 448 cm^−1^, non-bridging bending vibrations for O-Si-O can be found with the participation of aluminum, which can also substitute the spodumene structure in an octahedral position (Figure 8). The latter oscillations are also affected by the position of lithium (448 cm^−1^), which, combined with oxygen, contributes to their modification. Through a comparison with the crystals of jadeite, it can be seen that substitution with Na cation with an ionic radius of 186 pm [75] in the M6 position shifts these vibrations to 455 cm^−1^. In comparison, magnesium enstatite, in the M6 position, has 447 cm^−1^ vibrations (enstatite [76] and bronzite [77]). Lithium is a much lighter element than sodium, as its molar mass is 6.941 (for sodium it is 22.989 [51,78] close parenthesis g/mol) and its ionic radius is 152 pm. The full width at half maximum of the 455 cm^−1^ vibrations suggests that these vibrations are partly derived from the sodium at this position.

**Figure 9:**
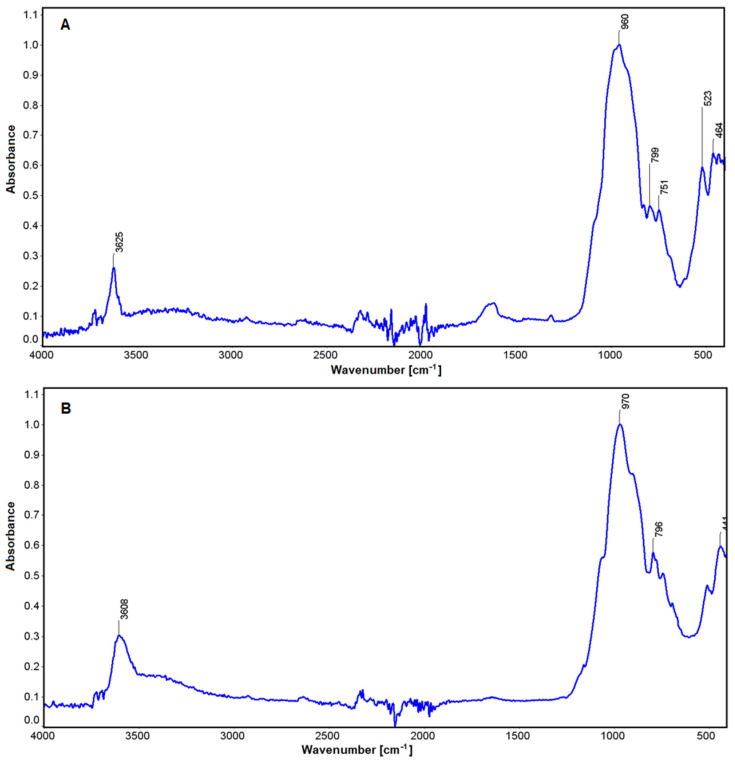
IR spectra for lepidolite (**A**) and fuchsite (**B**).

In the case of the study micas (fuchsite and lepidolite, Figure 9), comparisons were made with muscovite [79]. In both cases, the absorbance characteristic of stretching vibrations of the OH groups in the vicinity of 3625 and 3608 cm^−^^1^ is visible (Figure 9). These are determined by vibrations between ions located in octahedral groups and their interaction with water [80,81]. These differences become apparent depending on the nature of the ions in the analyzed minerals (Li in lepidolite, Cr in fuchsite). There is also a slight increase in absorbance in the region of 1621 cm^−^^1^, which is more pronounced for lepidolite. Another oscillation in the region of 970 cm^−^^1^ and 960 cm^−^^1^ is related to deformations produced through connection between aluminum and the OH group in these minerals [82]. In the region of 796–799 cm^−^^1^, there are Si-O deformations in both mica, and those at 750 cm^−^^1^ are characteristic of O-Al-O stretching vibrations in the tetrahedral position. Similarly, in lepidolite, a band in the 523 cm^−^^1^ region was found that is characteristic of Al(Li)-O-Si vibrations. The last absorbance in the 464 cm^−^^1^ and 441 cm^−^^1^ is associated with vibrations in the Si-O-Si group [82].

### 4.5. Single Crystals and XPS Analysis Results

#### 4.5.1. Fuchsite

**Figure 10:**
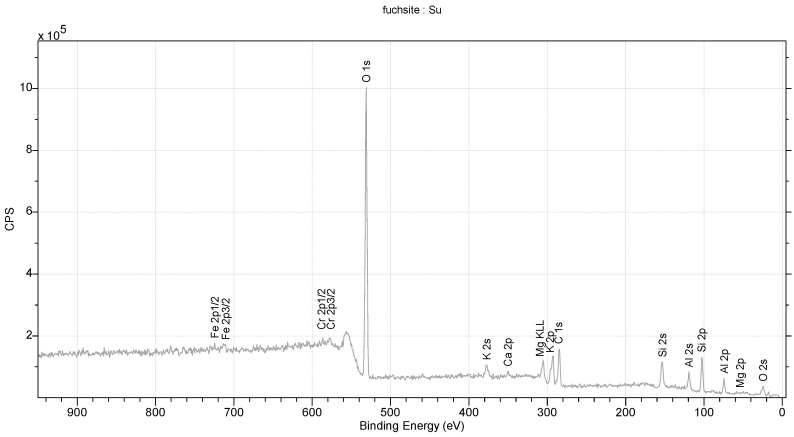
XPS spectrum for fuchsite.

**Figure 11:**
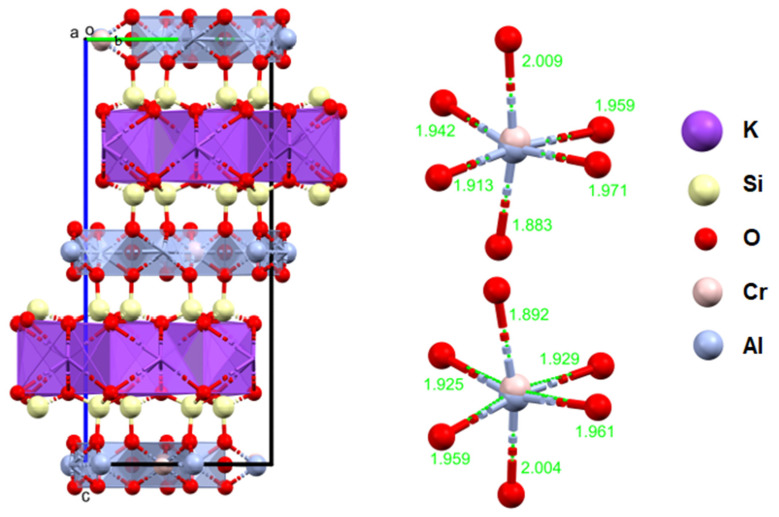
Crystal structure of the fuchsite from the analyzed rocks, as received by the authors (a, b, c—the main structural axes).

The studied fuchsite crystals, like all minerals in this group, contain chromium [82,83]. This is visible in the results of the diffraction measurements for the monocrystals and XPS. The X-ray data indicate that the studied fuchsite crystal contains 0.4 ions of this element per elemental cell of the crystal in the Cr-Al layer (Figure 7). It is worth noting the staggered position of both the Al^3+^ and Cr^3+^ ions at a distance of 0.12(1) Å (Figure 10 and Figure 11). This spread is due to the difference in the ionic radii of these two elements. The diffraction data show that only one position in the lattice, where the Al^3+^ ions are present, is occupied by additional Cr^3+^ ions, while the other position is 100% occupied by Al^3+^ ions (Figure 10). In addition, the layer has a free space filled, in this case, with water or an OH^-^ group in the amount of 0.1 molecule/ion per elementary cell. In this case, the OH^-^ group can compensate for the positive charge of the cations. In the Si-K layer, no admixture of other ions is observed at the detection level of this technique. All cell parameters and compositions are within the typical limits for this type of mineral. The thermal vibration ellipsoids observed in the experiment at 296 K had small thermal vibration amplitudes of U_iso_ 0.02–0.03 (Figure 11). This indicates strong interactions between ions in the lattice.

#### 4.5.2. Lepidolite

**Figure 12:**
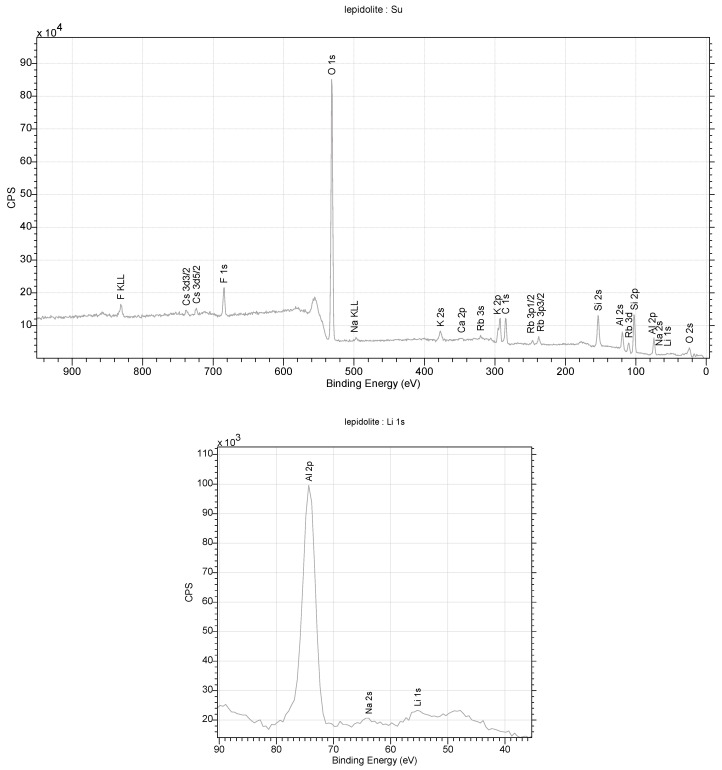
XPS spectra obtained for lepidolite from the studied rocks.

**Figure 13:**
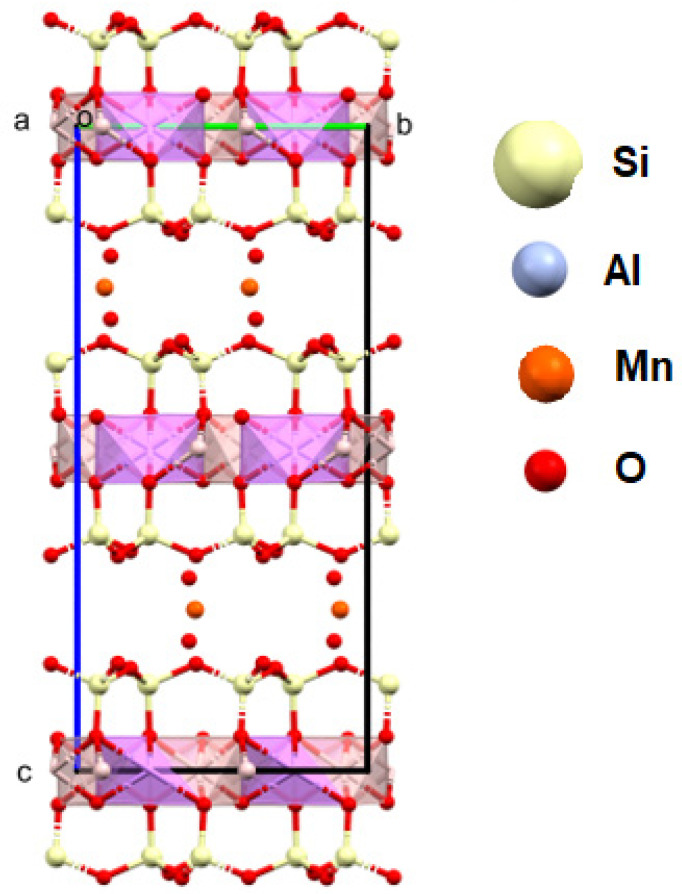
Crystal structure of lepidolite from the studied lithologies (a, b, c—the main structural axes).

Diffraction studies of the lepidolite yielded its structure [84,85,86,87]. They confirmed that it is a mineral from the silicate cluster, classified as lithium micas with an admixture of Fe^2+^ or Mn^2+^ in one position. However, unequivocally determining which ion is in this position is impossible with this technique. Positively charged Li-K ions reside in the space between the negatively charged aluminosilicate layers. In addition, OH^−^ group or water molecules in the amount of 0.1 per elementary cell of the crystal can be found in this crystal net space (Figure 12). In an elementary cell, there are three such layers in the direction of *b*. A richer atomic composition is provided by the data obtained through XPS measurements. This technique is more sensitive to heavier elements and allows for the unambiguous determination of their type. In contrast, lithium ions, which are difficult to determine via XPS, are visible in the monocrystalline structure. The ratio of K/Li ions is 2:1 in the structure. A close fit of the X-ray data with the model indicates that the lattice occupancy of Al^3+^ ions is 90 ± 5%. This indicates that aluminum shares this position with lighter ions, e.g., Mg and Na. However, (Figure 10), in this case, it is necessary to properly balance the charges in the lattice. Also, the occupancy of the Li position is less than 100%, which, in this case, may indicate the partial substitution of this position with water molecules. The low values of the Q_izo_ parameters (0.02–0.03) for atoms at 296 K indicate strong interactions between atoms (Figure 13).

#### 4.5.3. Spodumene

**Figure 14:**
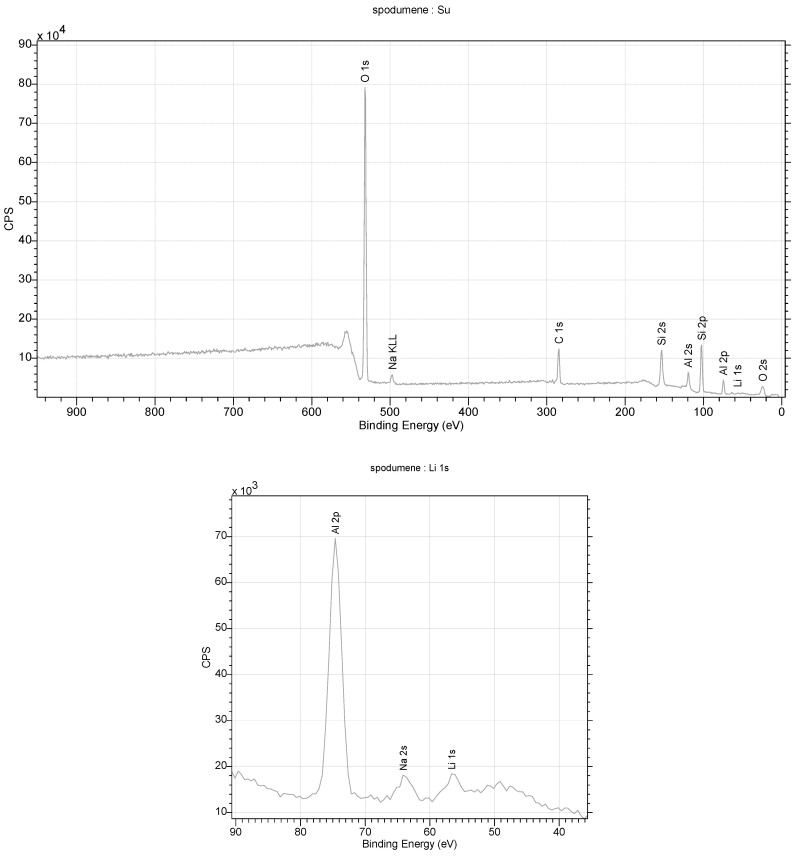
XPS spectra were obtained for spodumene from the studied rocks.

**Figure 15:**
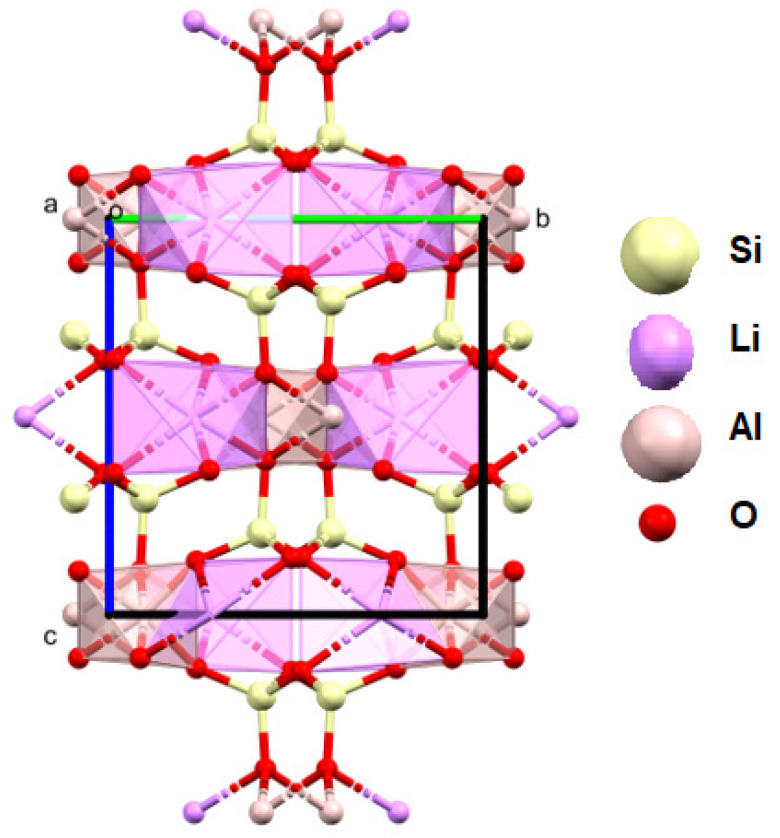
Crystal structure of spodumene (a, b, c—the main structural axes).

This is another studied mineral from the silicate cluster containing lithium [88,89,90,91]. In this case, the structural studies of the crystals indicated that the stoichiometric contents of Li and Al in the crystal lattice are within the error margin. Attempts to fit a crystal lattice model with a free occupancy parameter for Li indicated that the modeled electron density at this site is slightly higher than 1, at 1.06 (Figure 14). This may indicate a small content of a heavier element at this site, e.g., sodium, as suggested by the XPS and FTIR measurements. In the studied monocrystals, other ions were not visible in the crystal lattice (Figure 12). In the spodumene, silicate ions connect adjacent layers, so there is not enough space for additional ions between them, which explains the composition of this mineral. The compact lattice structure also contributes to strong interactions between atoms, which, in turn, manifests itself in the lowest Q_izo_ values of all the minerals presented (0.006–0.017) and its highest relative hardness (Figure 15).

## 5. Discussion

The minerals included in rocks found in northern Scandinavia (within Norway, Finland, and Russia) [92,93,94,95,96,97] were studied. These are exposed in many places in the area under discussion. Due to their mineralization, they may be significant in terms of raw material development, although the relatively small size of these pegmatite veins makes their profitability highly dependent on raw material prices. The mineralization of these pegmatites is the result of the crystallization of residual melts, which contain many incompatible elements [55,98]. On the other hand, the presence of some elements is related to the chemistry of the host rocks. This is particularly evident in the case of fuchsite, in which the presence of chromium may be related to specific rock types. Such small occurrences of fuchsite have been found by the authors in vein rocks in the Monchepluton area (Russia). Lepidolite and spodumene, on the other hand, show small admixtures of sodium, manganese, and iron, which may also be related to the rocks near these pegmatites. This was confirmed by both micro-area studies and monocrystal and XPS analyses. The presence of water in the mica and even in the spodumene (found using FTIR) confirms the hydrothermal nature of the association between these minerals [99,100]. Their nature in the discussed rocks varies. Optical studies indicated that the spodumene in the analyzed pegmatites usually has hypidiomorphic crystals, which may indicate crystallization as an early silicate phase. The presence of small admixtures of sodium may indicate that the mineral originally formed at a great depth, where higher pressures prevail, and then tectonically dislocated with solutions to its present site of occurrence, where it was hydrothermally altered [53,54,57]. On the other hand, aggregates of fuchsite and lepidolite tend to be secondary, co-occurring with other micas and occupying a position in the interstices of existing minerals; moreover, lepidolite can probably be a secondary mineral, formed at the expense of spodumene, as evidenced by our observations of this mineral in pegmatites. It is noteworthy that in addition to these minerals, there are many accessory phases, such as apatite and zircon. Alongside these, opaque minerals are present in large numbers. The presence of tin minerals points to the granitoid association as the source material for the origin of the schists [101,102]. In the case of the LCT pegmatites found in the Kaustinen area, these include, in addition to the aforementioned cassiterite and barite, magnetite, ilmenite, and titanite, as well as galena, sphalerite, and chalcopyrite. The presence of these minerals may also be related to the granitoid products and the action of hydrothermal products. The mineralization of Kolmozero pegmatites is also associated with the action of hydrothermal formations. This is evidenced by the mutual relations of rock-forming minerals with the observed bixbite, clarkeite, and sillenite, which are also accompanied by magnetite. Columbite and tantalite were also found in the LCT pegmatites.

## 6. Conclusions

The studied minerals are from selected rocks that are exposed in northern Scandinavia. The studied mineralization indicates the hydrothermal nature of these components. The studied minerals, including spodumene, lepidolite, and fuchsite, were formed after residual crystallization. The small admixtures of sodium present in the spodumene may indicate that it was formed under high pressures and, together with the melt, reached its present location, where it was altered due to pressure from hydrothermal fluids. The occurring lepidolite crystallized at a later stage, at the expense of spodumene, as evidenced by the structure of this mineral in the rock and by association (the occurrence of relics of spodumene in the vicinity of lepidolite). Fuchsite, like lepidolite, crystallized in the final stage, co-occurring with muscovite and biotite. The presence of chromium ions in this fuchsite is probably due to the occurrence of chromium-containing rocks in the vicinity of the formation of intrusions from the residual melts in which the studied rocks crystallized. The presence of accessory and opaque minerals also attests to the granitoid association of the original solutions. The minerals present may be of economic importance. Thorough research may contribute to the discovery of new locations of this type of pegmatite in the discussed area.

## Figures and Tables

**Table 1:** Single crystals’ X-ray diffraction results for all studied minerals.

Mineral	Fuchsite	Lepidolite	Spodumene
Empirical formula	Al_1.63_Cr_0.37_KSi_4_O_12.1_	Al_2_Fe_0.93_H_0.25_LiSi_4_O_12.12_	Al_2_Li_2_Si_4_O_12_
Temperature/K	294	294	294
Crystal system	monoclinic	monoclinic	monoclinic
Space group	C2/c	C2/c	I2/a
a/Å	5.2149(1)	5.2037(2)	5.2204(1)
b/Å	9.0505(2)	9.0250(4)	8.3947(2)
c/Å	20.0457(5)	20.1588(8)	9.0993(2)
α/°	90	90	90
β/°	95.78(1)	95.52(1)	102.40(1)
γ/°	90	90	90
Volume/Å^3^	941.29(4)	942.32(7)	389.462(17)
Z	4	4	2
ρ_calc_g/cm^3^	2.881	2.956	3.174
Crystal size/mm^3^	0.6 × 0.6 × 0.08	0.4 × 0.3 × 0.05	0.3 × 0.2 × 0.1
Data/restraints/parameters	844/0/89	854/0/98	344/0/48
Goodness-of-fit on F^2^	1.081	1.111	1.113
Final R indexes [I ≥ 2σ (I)]	R_1_ = 0.0530, wR_2_ = 0.1444	R_1_ = 0.0712, wR_2_ = 0.2039	R_1_ = 0.0184, wR_2_ = 0.0553
Largest diff. peak/hole/e Å^−3^	0.88/−0.79	0.88/−1.40	0.28/−0.26

## Appendix A

**Table A1:** Results of the SEM-EDS analysis of the mica minerals (wt.%).

Point *	O	F	Na	Mg	Al	Si	K	Ti	Cr	Fe	Mineral
Kautokeino(2)_pt9	38.47			5.78	6.13	28.90	9.29			6.82	biotite
Kautokeino(3)_pt2	40.41			1.26	13.50	30.67	10.79			1.20	biotite
Kautokeino(3)_pt3	39.56			1.12	13.85	30.65	10.80			1.01	biotite
Kautokeino(3)_pt10	39.30			6.03	6.94	33.30	9.03			3.66	biotite
Kautokeino(3)_pt11	38.96			5.56	7.56	32.41	9.13			4.77	biotite
Kautokeino(4)_pt7	39.52			6.69	7.10	30.44	10.85			3.54	biotite
Kautokeino(4)_pt8	38.51			6.97	6.57	30.13	11.49			4.41	biotite
Kautokeino(4)_pt9	38.67			7.13	8.05	28.56	10.28			5.04	biotite
Kautokeino(4)_pt10	37.87			7.31	7.68	29.72	9.70			6.34	biotite
Kautokeino(4)_pt11	38.60			6.61	8.07	28.23	10.15	2.34		4.05	biotite
Kautokeino(4)_pt13	38.19			6.57	7.59	29.19	9.72			7.07	biotite
Kautokeino(2)_pt8	31.59			5.03	5.07	24.08	6.89		4.66	13.44	fuchsite
Kautokeino(3)_pt1	39.04			0.95	12.15	29.82	9.90		5.99		fuchsite
Kautokeino(3)_pt4	38.06			0.90	11.74	28.50	10.21		7.75		fuchsite
Kautokeino(3)_pt5	37.84			1.10	12.46	28.46	10.10		6.51		fuchsite
Kautokeino(3)_pt6	38.17			1.10	12.66	29.35	9.94		5.9		fuchsite
Kautokeino(3)_pt7	37.02	1.31		1.01	12.65	26.91	10.73		6.78		fuchsite
Kautokeino(3)_pt8	37.61			1.20	12.41	29.77	10.08		6.36		fuchsite
Kautokeino(3)_pt9	38.06			1.07	12.13	31.38	9.01		6		fuchsite
Kautokeino(3)_pt16	39.94				1.77	46.38	2.21		6.43		fuchsite
Kautokeino(4)_pt12	36.05			5.94	6.70	24.05	9.14	2.13	3.96	10.16	fuchsite
Kautokeino(4)_pt14	34.89			6.63	6.27	26.51	7.25		5.93	11.10	fuchsite
Kautokeino(4)_pt15	35.44			5.67	6.18	27.83	8.58		5.42	9.02	fuchsite
Kautokeino(4)_pt16	36.18			2.54	10.94	27.54	9.90		5.14	6.18	fuchsite
Kautokeino(5)_pt12	36.14		0.73		8.64	34.58	13.82		4.5		fuchsite
Kautokeino(6)_pt4	37.66		0.61		7.75	35.16	12.63		4.54		fuchsite
Kautokeino(3)_pt17	42.47				1.99	50.46	2.88				muscovite
Kautokeino(3)_pt18	42.54				1.85	51.38	2.56				muscovite
Kolmozero(1)_pt1	39.02		0.83		17.38	27.63	9.12			0.44	muscovite
Kolmozero(1)_pt2	37.31		2.10		9.68	34.90	11.99				muscovite
Kolmozero(3)_pt1	37.17				15.63	25.25	11.08				muscovite
Kolmozero(3)_pt2	38.20		0.83		17.39	23.47	11.12				muscovite
Kolmozero(3)_pt3	38.89		0.72		18.13	24.77	12.08				muscovite
Kolmozero(3)_pt4	39.11		0.98		18.57	24.77	11.22				muscovite
Kolmozero(3)_pt5	39.22		0.85		18.01	25.55	11.69				muscovite
Kolmozero(3)_pt6	38.21		0.73		19.43	26.56	10.50				muscovite
Kolmozero(3)_pt7	39.77				18.76	26.84	10.23				muscovite
Kolmozero(4)_pt2	38.25				17.17	26.68	11.62				muscovite
Kolmozero(4)_pt3	38.44				17.68	24.78	10.67				muscovite
Kolmozero(5)_pt4	37.23				9.39	32.79	13.13				muscovite
Kolmozero(8)_pt9	39.78				15.22	31.53	9.76				muscovite
Kolmozero(8)_pt10	39.89				16.61	31.13	9.80				muscovite
Kolmozero(8)_pt11	41.80				9.40	39.63	6.02				muscovite
Kaustinen(3)_pt1	39.27				13.18	29.66	8.86				muscovite
Kaustinen(3)_pt4	41.17				16.21	30.53	7.95				muscovite
Kaustinen(3)_pt5	40.52				16.38	29.32	9.09				muscovite
Kaustinen(3)_pt6	41.54		0.47		17.40	30.99	7.62				muscovite
Kaustinen(4)_pt2	39.23				13.48	29.10	8.63				muscovite
Kaustinen(4)_pt3	39.95				14.02	27.82	9.82				muscovite
Kaustinen(4)_pt4	40.15				13.17	30.72	9.08				muscovite
Kaustinen(4)_pt5	40.33				14.35	29.98	8.70				muscovite
Kaustinen2(2)_pt3	36.97		1.55		10.25	32.17	17.82				muscovite
Kaustinen2(2)_pt4	37.19		1.04		10.47	32.31	17.49				muscovite
Kolmozero(18)_pt1	40.37				18.81	28.01	11.61				muscovite
Kolmozero(18)_pt2	39.06		0.47		19.48	27.32	10.97				muscovite
Kolmozero(18)_pt3	39.58				19.91	26.77	11.55				muscovite
Kolmozero(18)_pt4	39.57				19.37	26.44	12.23				muscovite
Kolmozero(18)_pt5	39.48				20.47	26.04	11.26				muscovite
Kolmozero(18)_pt6	37.54		0.68		18.20	25.47	10.24				muscovite
Kolmozero(20)_pt5	40.13				15.57	31.96	8.82				muscovite
Kolmozero(20)_pt6	40.33				15.65	30.28	8.94				muscovite
Kolmozero(20)_pt8	39.69			0.18	15.94	30.06	10.06				muscovite
Kaustinen(10)_pt8	41.26				17.87	26.38	7.57				muscovite
Kaustinen(10)_pt9	41.38				16.28	25.38	7.66		2.67		muscovite
Kolmozero2(4)_pt1	32.04	2.29	0.29		12.96	23.21	7.85				lepidolite
Kolmozero2(4)_pt2	33.98	3.32			13.57	24.75	8.64				lepidolite
Kolmozero2(4)_pt3	34.25	3.14	0.37		13.64	25.03	8.52				lepidolite
Kolmozero2(4)_pt4	34.65	2.89	0.35		14.11	25.93	8.68				lepidolite
Kolmozero2(4)_pt5	34.63	2.93	0.59	0.12	14.42	26.26	8.58				lepidolite
Kolmozero2(4)_pt6	35.52	3.76			15.25	29.76	9.38				lepidolite
Kolmozero2(4)_pt7	35.47	3.34	0.49		15.17	29.82	10.43				lepidolite
Kolmozero2(4)_pt8	35.30	3.09	0.52		14.63	29.40	9.66				lepidolite
Kolmozero2(4)_pt9	33.58	2.99	0.48		14.51	27.44	9.17				lepidolite
Kolmozero2(4)_pt10	34.99	3.47		0.44	14.05	27.38	8.28				lepidolite
Kolmozero2(4)_pt11	33.49	0.22	0.62		10.57	31.00	12.48				lepidolite
Kolmozero2(4)_pt12	32.11	0.51	0.44		10.53	31.61	13.20				lepidolite
Kolmozero2(4)_pt13	34.31	0.51	0.30		10.81	31.96	12.31				lepidolite
Kolmozero2(4)_pt14	33.98	0.68			11.21	32.59	13.15				lepidolite
Kolmozero2(4)_pt15	35.65	3.42			14.99	29.24	9.89				lepidolite
Kolmozero2(5)_pt1	33.59	2.55			13.54	25.85	8.51				lepidolite
Kolmozero2(5)_pt2	34.15	3.54			13.23	26.37	9.10				lepidolite
Kolmozero2(5)_pt3	33.72	3.68	0.30		14.24	28.20	8.37				lepidolite
Kolmozero2(5)_pt4	34.71	3.62			14.57	27.89	9.68				lepidolite
Kolmozero2(5)_pt5	34.30	5.37			11.93	30.88	9.34				lepidolite
Kolmozero2(5)_pt6	34.42	0.92			11.49	32.68	13.07				lepidolite
Kolmozero2(5)_pt7	34.20	0.69	0.32		11.44	32.66	14.01				lepidolite
Kolmozero2(5)_pt8	34.69	3.76			15.66	29.86	10.63				lepidolite
Kolmozero2(5)_pt9	35.97	3.36			15.35	30.02	10.31				lepidolite
Kolmozero2(5)_pt10	36.15	3.60			15.83	30.36	10.08				lepidolite
Kolmozero2(5)_pt11	35.81	3.15			15.74	30.51	10.43				lepidolite
Kolmozero2(6)_pt4	41.35	0.52	0.83		15.98	33.98	3.64				lepidolite
Kolmozero2(2)_pt5	36.38		0.71		12.94	34.83	14.26				lepidolite
Kolmozero2(1)_pt7	37.96		0.34		12.15	35.65	12.84				lepidolite

* In this analysis, there is no possibility of lithium indication.

**Table A2:** Results of the SEM-EDS of spodumene (wt.%).

Point *	C	O	F	Na	Mg	Al	Si	Mineral
Kaustinen(3)_pt14	2.67	44.24				15.82	37.26	spodumene
Kaustinen(3)_pt15	2.84	44.21				15.79	37.15	spodumene
Kaustinen(3)_pt16	2.47	45.12				15.86	36.55	spodumene
Kolmozero(2)_pt1	6.72	39.68		6.48		11.17	35.95	spodumene
Kolmozero(2)_pt2	5.75	40.09		6.67		11.53	35.96	spodumene
Kolmozero(2)_pt4	2.82	40.45		7.15		11.79	37.78	spodumene
Kolmozero(2)_pt5	3.49	39.73		7.17		11.74	37.86	spodumene
Kolmozero(3)_pt8	2.65	39.69		7.09		11.35	37.51	spodumene
Kolmozero(3)_pt9	3.76	39.19		7.51		11.72	36.94	spodumene
Kolmozero(3)_pt10	3.11	40.44		6.20		11.34	37.27	spodumene
Kolmozero(3)_pt11	3.59	42.46		1.52		14.92	36.26	spodumene
Kolmozero(3)_pt12	4.39	41.44		4.15		11.18	38.84	spodumene
Kolmozero(3)_pt13	5.01	40.19		1.22		9.79	37.67	spodumene
Kolmozero(5)_pt3	4.80	43.59				14.07	37.54	spodumene
Kolmozero(7)_pt4	7.68	42.37		0.66		14.02	35.27	spodumene
Kolmozero(7)_pt5	5.80	43.61				14.07	36.52	spodumene
Kolmozero(8)_pt1	14.42	40.36				12.57	32.66	spodumene
Kolmozero(8)_pt2	16.24	41.07				12.94	29.75	spodumene
Kolmozero(8)_pt6	6.92	39.78				1.19	52.11	spodumene
Kolmozero(8)_pt7	4.70	41.89				1.17	52.24	spodumene
Kolmozero(8)_pt8	2.64	42.38				1.13	53.84	spodumene
Kolmozero(8)_pt12	2.65	43.13				2.01	52.21	spodumene
Kolmozero(9)_pt1	17.23	41.85				12.10	28.81	spodumene
Kolmozero(9)_pt2	10.68	40.60		4.01		11.51	33.19	spodumene
Kolmozero(9)_pt3	9.61	42.79				4.71	42.89	spodumene
Kolmozero(9)_pt4	7.71	44.53				11.75	36.01	spodumene
Kolmozero(9)_pt5	55.02	33.59				2.18	9.21	spodumene
Kolmozero(11)_pt7	4.89	42.72		1.77		14.79	35.83	spodumene
Kolmozero(11)_pt8	4.16	40.72		6.32		11.18	37.62	spodumene
Kolmozero(11)_pt9	4.30	41.67		7.35		11.37	35.31	spodumene
Kolmozero(11)_pt10	3.30	41.02		7.36		11.50	36.82	spodumene
Kolmozero(11)_pt11	3.15	39.99		8.01		11.21	37.64	spodumene
Kolmozero(12)_pt15	6.47	39.50		7.58		10.99	35.46	spodumene
Kolmozero(12)_pt16	6.16	39.91		7.36		11.33	35.24	spodumene
Kolmozero(12)_pt17	5.89	39.88		7.46		10.24	36.53	spodumene
Kolmozero(13)_pt1	3.25	40.25		8.04		10.36	38.10	spodumene
Kolmozero(14)_pt12	9.92	41.91				13.10	35.07	spodumene
Kolmozero(14)_pt13	7.61	40.92		0.55		2.33	48.58	spodumene
Kolmozero(14)_pt14	7.08	41.82				1.82	49.28	spodumene
Kolmozero(16)_pt1	16.26	40.72				13.29	29.73	spodumene
Kolmozero(16)_pt2	22.45	38.80				10.95	27.79	spodumene
Kolmozero(16)_pt3	14.29	42.10				13.22	30.39	spodumene
Kolmozero(16)_pt4	15.08	40.91				12.61	31.40	spodumene
Kolmozero(16)_pt5	14.35	39.36				12.14	27.78	spodumene
Kolmozero(16)_pt6	16.22	41.32				13.21	29.25	spodumene
Kolmozero(16)_pt7	48.29	33.01				4.66	11.81	spodumene
Kaustinen(2)_pt1	3.10	44.54				14.12	38.25	spodumene
Kaustinen(2)_pt2	2.78	44.26				15.39	37.57	spodumene
Kaustinen(2)_pt3	2.73	43.36				14.12	35.14	spodumene
Kaustinen(2)_pt4	3.25	45.14				13.73	37.88	spodumene
Kaustinen(2)_pt5	2.40	45.10				14.38	38.11	spodumene
Kaustinen(2)_pt6	1.98	42.98				4.05	50.99	spodumene
Kaustinen(1)_pt4	15.22	39.54				0.75	44.49	spodumene
Kaustinen(1)_pt5	16.39	39.16					41.13	spodumene
Kaustinen(1)_pt6	16.95	39.84					43.22	spodumene
Kaustinen(1)_pt7	19.24	38.76				0.53	41.47	spodumene
Kaustinen(1)_pt8	14.67	40.34				0.63	44.37	spodumene
Kaustinen(1)_pt9	12.67	40.84					46.48	spodumene
Kaustinen(3)_pt2	10.36	41.85				1.67	43.23	spodumene
Kaustinen(3)_pt3	12.06	41.64				1.69	44.61	spodumene
Kaustinen(3)_pt7	2.89	44.46				12.91	39.75	spodumene
Kaustinen(3)_pt8	3.14	43.49				12.69	40.68	spodumene
Kaustinen(3)_pt10	3.82	45.32				15.39	35.47	spodumene
Kaustinen(6)_pt6	2.24	44.68				2.72	50.35	spodumene
Kaustinen(7)_pt1	2.34	44.62		0.88		14.73	37.43	spodumene
Kaustinen(7)_pt2	2.45	44.46		0.84		15.01	37.25	spodumene
Kaustinen(7)_pt3	2.63	43.62		0.73		14.15	38.87	spodumene
Kaustinen(7)_pt4	3.73	46.01		0.69		13.10	36.48	spodumene
Kaustinen(7)_pt8	2.81	43.03				1.70	52.46	spodumene
Kaustinen(7)_pt9	3.89	41.11				5.03	44.12	spodumene
Kolmozero(17)_pt1	16.85	41.72		1.70		11.72	28.01	spodumene
Kolmozero(17)_pt2	17.95	43.16				11.50	27.39	spodumene
Kolmozero(17)_pt3	13.63	39.68				13.97	30.33	spodumene
Kolmozero(17)_pt4	7.19	41.22				15.74	35.84	spodumene
Kolmozero(17)_pt5	4.63	44.85		0.56		14.39	35.58	spodumene
Kolmozero(17)_pt6	4.69	41.82		0.98		15.42	37.09	spodumene
Kolmozero(17)_pt7	2.60	44.11		0.58		16.02	36.70	spodumene
Kolmozero(17)_pt8	2.61	43.70				16.19	37.50	spodumene
Kolmozero(18)_pt15	4.18	43.12				15.75	36.95	spodumene
Kolmozero(18)_pt16	2.24	44.51				16.80	36.44	spodumene
Kolmozero(19)_pt3	7.15	42.68				9.72	28.44	spodumene
Kolmozero(19)_pt4	2.59	44.00				2.27	51.14	spodumene
Kolmozero(19)_pt5	5.55	43.75				12.28	38.41	spodumene
Kolmozero(19)_pt6	5.57	42.40		0.89		13.59	37.54	spodumene
Kolmozero(19)_pt8	2.42	44.24				14.48	38.86	spodumene
Kolmozero(19)_pt9	5.11	42.75		0.60		14.68	36.87	spodumene
Kolmozero(20)_pt9	4.91	40.01		0.62		15.57	29.61	spodumene
Kolmozero(20)_pt10	5.44	43.93				13.23	36.00	spodumene
Kolmozero(20)_pt11	3.99	44.34				13.36	36.58	spodumene
Kolmozero(20)_pt12	4.33	43.88		0.79		13.11	36.20	spodumene
Kaustinen(10)_pt2	6.60	44.88				14.61	33.90	spodumene
Kaustinen(10)_pt3	4.51	45.00				15.49	35.00	spodumene
Kaustinen(10)_pt4	4.84	45.31				15.71	34.14	spodumene
Kaustinen(10)_pt5	5.32	44.41				15.05	35.22	spodumene
Kaustinen(10)_pt6	5.26	44.87				15.56	34.32	spodumene
Kaustinen(10)_pt7	4.64	44.56				16.47	34.32	spodumene
Kaustinen(13)_pt2	6.00	43.27		0.91		11.53	34.39	spodumene
Kaustinen(13)_pt3	5.63	44.74		0.97		12.24	36.42	spodumene
Kaustinen(13)_pt8	4.09	42.63		0.99		1.67	50.63	spodumene

* In this analysis there is no possibility of Lithium indicated.

**Table A3:** Fractional atomic coordinates (×10^4^) and equivalent isotropic displacement parameters (Å^2^ × 10^3^) for fuchsite. U_eq_ is defined as 1/3 of the trace of the orthogonalized U_IJ_ tensor.

Atom	*x*	*y*	*z*	U(eq)
K1	5000	4023.4(14)	7500	26.6(4)
Cr1	2370(20)	5812(8)	5015(5)	6(3)
Al2	2577(9)	5839(4)	4992(2)	13.2(16)
Si2	9645.9(19)	5705.7(11)	6358.7(5)	12.0(4)
Si1	4518.5(19)	7416.3(10)	6358.9(5)	11.8(4)
O2	3888(5)	7483(2)	5535.9(13)	14.9(7)
O4	9591(5)	5591(3)	5537.9(13)	14.7(6)
O6	9245(6)	4067(3)	6685.3(13)	17.8(7)
O1	5445(6)	5637(3)	4491.4(13)	16.4(6)
O5	12.457(5)	6332(3)	6690.0(13)	18.0(7)
O3	7455(5)	6852(3)	6587.4(13)	19.7(7)
O7	7500	7500	5000	7(13)

**Table A4:** Anisotropic displacement parameters (Å^2^ × 10^3^) for fuchsite. The anisotropic displacement factor exponent takes the form: −2π^2^[h^2^a * ^2^U_11_ + 2hka * b * U_12_+…].

Atom	U_11_	U_22_	U_33_	U_23_	U_13_	U_12_
K1	27.8(8)	26.1(8)	25.7(7)	0	2.3(5)	0
Si2	13.5(7)	8.8(6)	13.6(6)	−0.2(3)	1.3(4)	−0.2(3)
Si1	13.2(7)	9.9(6)	12.2(6)	0.4(3)	0.5(4)	0.0(3)
O2	17.0(16)	11.9(13)	15.6(14)	−1.9(8)	0.3(11)	−1.5(9)
O4	15.5(14)	11.9(12)	16.1(14)	0.3(10)	−0.5(9)	−2.1(10)
O6	26.3(16)	12.5(14)	15.0(13)	−0.9(9)	4.0(11)	−1.6(10)
O1	19.8(15)	15.2(13)	14.4(13)	2.8(10)	2.9(10)	2.3(10)
O5	21.3(15)	16.4(14)	16.1(13)	0.8(10)	0.5(11)	−4.8(11)
O3	19.3(15)	20.3(15)	18.9(13)	−4.3(10)	−0.7(10)	4.4(11)

**Table A5:** Bond lengths for fuchsite.

Atom	Atom	Length/Å	Atom	Atom	Length/Å
K1	Si1 ^1^	3.7400(10)	Cr1	O1 ^10^	1.943(10)
K1	Si1 ^2^	3.7400(10)	Cr1	O1	2.010(11)
K1	O6 ^3^	2.881(3)	Al2	Al2 ^10^	2.946(9)
K1	O6 ^4^	3.269(3)	Al2	O2	1.928(5)
K1	O6 ^5^	3.269(3)	Al2	O2 ^9^	1.962(5)
K1	O6	2.881(3)	Al2	O4 ^10^	1.960(5)
K1	O5 ^4^	2.885(3)	Al2	O4 ^5^	2.004(6)
K1	O5 ^6^	3.261(3)	Al2	O1	1.893(5)
K1	O5 ^7^	3.261(3)	Al2	O1 ^10^	1.924(5)
K1	O5 ^5^	2.885(3)	Si2	O4	1.646(3)
K1	O3 ^6^	2.912(3)	Si2	O6	1.643(3)
K1	O3 ^7^	2.912(3)	Si2	O5	1.648(3)
Cr1	Cr1 ^8^	2.87(2)	Si2	O3	1.642(3)
Cr1	O2	1.960(9)	Si1	O2	1.650(3)
Cr1	O2 ^9^	1.970(9)	Si1	O6 ^11^	1.643(2)
Cr1	O4 ^5^	1.882(11)	Si1	O5 ^5^	1.644(3)
Cr1	O4 ^10^	1.911(9)	Si1	O3	1.635(3)

^1^ 1/2-X,-1/2 + Y,3/2-Z; ^2^ 1/2 + X,-1/2 + Y,+Z; ^3^ 1-X,+Y,3/2-Z; ^4^ 2-X,+Y,3/2-Z; ^5^ -1 + X,+Y,+Z; ^6^ -1/2 + X,-1/2 + Y,+Z; ^7^ 3/2-X,-1/2 + Y,3/2-Z; ^8^ -X,1-Y,1-Z; ^9^ 1/2-X,3/2-Y,1-Z; ^10^ 1-X,1-Y,1-Z; ^11^ -1/2 + X,1/2 + Y,+Z.

Si1 ^1^	K1	Si1 ^2^	134.23(4)	O2	Al2	O4 ^10^	165.5(3)
O6 ^3^	K1	Si1 ^2^	24.63(5)	O2 ^8^	Al2	O4 ^5^	95.7(2)
O6 ^4^	K1	Si1 ^1^	72.96(5)	O4 ^10^	Al2	Al2 ^10^	96.5(2)
O6 ^5^	K1	Si1 ^1^	107.60(5)	O4 ^5^	Al2	Al2 ^10^	130.5(3)
O6	K1	Si1 ^1^	24.63(5)	O4 ^10^	Al2	O2 ^8^	92.7(2)
O6	K1	Si1 ^2^	156.87(6)	O4 ^10^	Al2	O4 ^5^	77.6(2)
O6 ^3^	K1	Si1 ^1^	156.87(6)	O1	Al2	Al2 ^10^	39.88(14)
O6 ^5^	K1	Si1 ^2^	72.96(5)	O1 ^10^	Al2	Al2 ^10^	39.10(13)
O6 ^4^	K1	Si1 ^2^	107.60(5)	O1	Al2	O2	96.9(2)
O6 ^3^	K1	O6	178.45(11)	O1	Al2	O2 ^8^	94.2(2)
O6	K1	O6 ^5^	115.84(8)	O1 ^10^	Al2	O2	95.3(2)
O6 ^3^	K1	O6 ^4^	115.84(8)	O1 ^10^	Al2	O2 ^8^	170.4(3)
O6 ^3^	K1	O6 ^5^	64.13(8)	O1 ^10^	Al2	O4 ^5^	91.9(2)
O6	K1	O6 ^4^	64.14(8)	O1	Al2	O4 ^10^	95.3(2)
O6 ^4^	K1	O6 ^5^	178.63(9)	O1	Al2	O4 ^5^	168.0(2)
O6 ^3^	K1	O5 ^5^	88.48(7)	O1 ^10^	Al2	O4 ^10^	94.7(2)
O6	K1	O5 ^6^	130.96(7)	O1	Al2	O1 ^10^	79.0(2)
O6 ^3^	K1	O5 ^7^	130.96(8)	K1 ^11^	Si2	K1	87.18(2)
O6 ^3^	K1	O5 ^6^	50.56(7)	K1 ^11^	Si2	K1 ^12^	88.01(2)
O6	K1	O5 ^7^	50.56(6)	K1 ^12^	Si2	K1	87.08(2)
O6 ^3^	K1	O5 ^4^	90.40(8)	O4	Si2	K1 ^12^	130.69(10)
O6	K1	O5 ^5^	90.40(8)	O4	Si2	K1 ^11^	121.42(10)
O6	K1	O5 ^4^	88.48(8)	O4	Si2	K1	128.79(10)
O6	K1	O3 ^7^	88.89(7)	O4	Si2	O5	110.47(14)
O6 ^3^	K1	O3 ^6^	88.89(7)	O6	Si2	K1	44.48(10)
O6 ^3^	K1	O3 ^7^	92.16(8)	O6	Si2	K1 ^12^	118.54(10)
O6	K1	O3 ^6^	92.16(8)	O6	Si2	K1 ^11^	60.47(11)
O5 ^4^	K1	Si1 ^1^	110.10(5)	O6	Si2	O4	110.57(14)
O5 ^5^	K1	Si1 ^1^	102.68(5)	O6	Si2	O5	107.07(15)
O5 ^5^	K1	Si1 ^2^	110.10(5)	O5	Si2	K1 ^12^	59.61(10)
O5 ^7^	K1	Si1 ^2^	108.51(6)	O5	Si2	K1	119.26(10)
O5 ^4^	K1	Si1 ^2^	102.68(6)	O5	Si2	K1 ^11^	46.85(10)
O5 ^6^	K1	Si1 ^2^	26.03(5)	O3	Si2	K1	65.35(11)
O5 ^7^	K1	Si1 ^1^	26.03(5)	O3	Si2	K1 ^11^	125.61(10)
O5 ^6^	K1	Si1 ^1^	108.51(6)	O3	Si2	K1 ^12^	47.15(9)
O5 ^6^	K1	O6 ^4^	98.47(7)	O3	Si2	O4	112.10(14)
O5 ^5^	K1	O6 ^5^	50.47(7)	O3	Si2	O6	109.83(15)
O5 ^7^	K1	O6 ^4^	82.56(6)	O3	Si2	O5	106.61(14)
O5 ^6^	K1	O6 ^5^	82.56(6)	K1^12^	Si1	K1	86.95(2)
O5 ^7^	K1	O6 ^5^	98.47(7)	K1 ^13^	Si1	K1	87.36(2)
O5 ^4^	K1	O6 ^5^	128.25(7)	K1 ^13^	Si1	K1 ^12^	87.98(2)
O5 ^5^	K1	O6 ^4^	128.25(8)	O2	Si1	K1 ^13^	121.52(10)
O5 ^4^	K1	O6 ^4^	50.47(7)	O2	Si1	K1 ^12^	130.93(10)
O5 ^5^	K1	O5 ^7^	116.24(8)	O2	Si1	K1	128.47(9)
O5 ^5^	K1	O5 ^6^	129.78(2)	O6 ^13^	Si1	K1 ^13^	46.95(10)
O5 ^4^	K1	O5 ^7^	129.78(2)	O6 ^13^	Si1	K1 ^12^	59.88(11)
O5 ^4^	K1	O5 ^5^	87.22(11)	O6 ^13^	Si1	K1	119.68(10)
O5 ^4^	K1	O5 ^6^	116.24(8)	O6 ^13^	Si1	O2	110.39(13)
O5 ^7^	K1	O5 ^6^	83.32(9)	O6 ^13^	Si1	O5 ^5^	107.19(15)
O5 ^5^	K1	O3 ^7^	88.88(8)	O5 ^5^	Si1	K1	44.35(10)
O5 ^4^	K1	O3^6^	88.88(8)	O5 ^5^	Si1	K1 ^13^	60.49(10)
O5 ^4^	K1	O3 ^7^	175.28(8)	O5 ^5^	Si1	K1 ^12^	118.17(10)
O5 ^5^	K1	O3 ^6^	175.28(8)	O5 ^5^	Si1	O2	110.66(13)
O3 ^6^	K1	Si1 ^2^	68.20(6)	O3	Si1	K1 ^13^	125.45(10)
O3 ^7^	K1	Si1 ^1^	68.20(6)	O3	Si1	K1 ^12^	47.06(10)
O3 ^7^	K1	Si1 ^2^	81.17(6)	O3	Si1	K1	65.07(11)
O3 ^6^	K1	Si1 ^1^	81.17(6)	O3	Si1	O2	112.21(14)
O3 ^7^	K1	O6 ^5^	49.98(7)	O3	Si1	O6 ^13^	106.78(15)
O3 ^6^	K1	O6 ^4^	49.98(7)	O3	Si1	O5 ^5^	109.42(14)
O3 ^7^	K1	O6 ^4^	131.24(7)	Cr1	O2	Cr1 ^8^	102.2(3)
O3 ^6^	K1	O6 ^5^	131.24(7)	Cr1	O2	Al2 ^8^	101.2(4)
O3 ^6^	K1	O5 ^7^	68.39(7)	Si1	O2	Cr1	122.3(3)
O3 ^7^	K1	O5 ^7^	50.22(7)	Si1	O2	Cr1 ^8^	127.6(3)
O3 ^6^	K1	O5 ^6^	50.22(7)	Si1	O2	Al2	124.06(18)
O3 ^7^	K1	O5 ^6^	68.39(8)	Si1	O2	Al2 ^8^	126.55(19)
O3 ^7^	K1	O3 ^6^	95.13(11)	Cr1 ^11^	O4	Cr1 ^10^	98.3(4)
O2 ^8^	Cr1	Cr1 ^9^	98.8(6)	Cr1 ^11^	O4	Al2 ^11^	0.8(4)
O2	Cr1	Cr1 ^9^	135.0(7)	Cr1 ^10^	O4	Al2 ^11^	99.0(4)
O2	Cr1	O2 ^8^	77.8(3)	Cr1 ^10^	O4	Al2 ^10^	3.4(4)
O2 ^8^	Cr1	O1	90.4(4)	Cr1 ^11^	O4	Al2 ^10^	101.6(4)
O2	Cr1	O1	92.2(4)	Si2	O4	Cr1 ^10^	125.1(3)
O4 ^5^	Cr1	Cr1 ^9^	41.3(3)	Si2	O4	Cr1 ^11^	127.8(3)
O4 ^10^	Cr1	Cr1 ^9^	40.5(2)	Si2	O4	Al2 ^10^	122.33(19)
O4 ^10^	Cr1	O2	170.2(6)	Si2	O4	Al2 ^11^	127.0(2)
O4 ^10^	Cr1	O2 ^8^	93.9(5)	K1	O6	K1 ^11^	115.84(8)
O4 ^5^	Cr1	O2 ^8^	99.5(5)	Si2	O6	K1 ^11^	93.59(12)
O4 ^5^	Cr1	O2	94.4(5)	Si2	O6	K1	111.96(13)
O4 ^5^	Cr1	O4 ^10^	81.7(4)	Si1 ^1^	O6	K1	108.42(13)
O4 ^10^	Cr1	O1	93.0(4)	Si1 ^1^	O6	K1^11^	94.36(12)
O4 ^5^	Cr1	O1	169.1(5)	Si1 ^1^	O6	Si2	129.97(17)
O4 ^10^	Cr1	O1 ^10^	95.7(4)	Cr1 ^10^	O1	Cr1	104.3(4)
O4 ^5^	Cr1	O1 ^10^	95.1(4)	K1 ^11^	O5	K1 ^12^	116.24(8)
O1 ^10^	Cr1	Cr1 ^9^	97.1(4)	Si2	O5	K1 ^12^	94.54(12)
O1	Cr1	Cr1 ^9^	132.8(6)	Si2	O5	K1 ^11^	108.52(12)
O1 ^10^	Cr1	O2	93.6(5)	Si1 ^11^	O5	K1 ^11^	112.18(13)
O1 ^10^	Cr1	O2 ^8^	163.5(6)	Si1 ^11^	O5	K1 ^12^	93.48(11)
O1 ^10^	Cr1	O1	75.7(4)	Si1 ^11^	O5	Si2	129.33(16)
O2 ^8^	Al2	Al2 ^10^	133.8(3)	Si2	O3	K1 ^12^	108.42(12)
O2	Al2	Al2 ^10^	97.9(3)	Si1	O3	K1 ^12^	108.66(13)
O2	Al2	O2 ^8^	78.70(18)	Si1	O3	Si2	140.99(17)
O2	Al2	O4 ^5^	91.6(2)				
^1^ 1/2 + X,-1/2 + Y,+Z; ^2^ 1/2-X,-1/2 + Y,3/2-Z; ^3^ 1-X,+Y,3/2-Z; ^4^ 2-X,+Y,3/2-Z; ^5^ -1 + X,+Y,+Z; ^6^ 3/2-X,-1/2 + Y,3/2-Z; ^7^ -1/2 + X,-1/2 + Y,+Z; ^8^ 1/2-X,3/2-Y,1-Z; ^9^ -X,1-Y,1-Z; ^10^ 1-X,1-Y,1-Z; ^11^ 1 + X,+Y,+Z; ^12^ 1/2 + X,1/2 + Y,+Z; ^13^ -1/2 + X,1/2 + Y,+Z.							

**Table A6:** Torsion angles for fuchsite.

A	B	C	D	Angle/˚	A	B	C	D	Angle/˚
K1 ^1^	Si2	O4	Cr1 ^1^	−41.6(4)	O4	Si2	O6	K1 ^1^	115.30(12)
K1	Si2	O4	Cr1 ^1^	−156.3(3)	O4	Si2	O6	K1	−124.95(13)
K1 ^2^	Si2	O4	Cr1 ^1^	76.5(4)	O4	Si2	O6	Si1 ^4^	16.6(3)
K1 ^2^	Si2	O4	Cr1 ^3^	−143.2(4)	O4	Si2	O5	K1 ^2^	126.00(11)
K1 ^1^	Si2	O4	Cr1 ^3^	98.6(4)	O4	Si2	O5	K1 ^1^	−114.38(13)
K1	Si2	O4	Cr1 ^3^	−16.0(4)	O4	Si2	O5	Si1 ^1^	27.7(3)
K1 ^1^	Si2	O4	Al2 ^3^	101.0(2)	O4	Si2	O3	K1 ^2^	−125.67(13)
K1	Si2	O4	Al2 ^3^	−13.7(3)	O4	Si2	O3	Si1	35.6(4)
K1 ^1^	Si2	O4	Al2 ^1^	−41.8(2)	O6	Si2	O4	Cr1 ^3^	31.5(4)
K1 ^2^	Si2	O4	Al2 ^1^	76.3(2)	O6	Si2	O4	Cr1 ^1^	−108.8(4)
K1 ^2^	Si2	O4	Al2 ^3^	−140.89(19)	O6	Si2	O4	Al2 ^3^	33.8(3)
K1	Si2	O4	Al2 ^1^	−156.50(17)	O6	Si2	O4	Al2 ^1^	−109.0(2)
K1	Si2	O6	K1 ^1^	−119.75(13)	O6	Si2	O5	K1 ^2^	−113.54(12)
K1 ^1^	Si2	O6	K1	119.75(13)	O6	Si2	O5	K1 ^1^	6.08(16)
K1 ^2^	Si2	O6	K1	50.46(15)	O6	Si2	O5	Si1 ^1^	148.2(2)
K1 ^2^	Si2	O6	K1 ^1^	−69.29(10)	O6	Si2	O3	K1 ^2^	111.00(14)
K1 ^1^	Si2	O6	Si1 ^4^	−98.7(3)	O6	Si2	O3	Si1	−87.7(3)
K1 ^2^	Si2	O6	Si1 ^4^	−168.03(18)	O6 ^5^	Si1	O2	Cr1 ^6^	−5.9(5)
K1	Si2	O6	Si1 ^4^	141.5(3)	O6 ^5^	Si1	O2	Cr1	−149.3(4)
K1	Si2	O5	K1 ^2^	−66.66(9)	O6 ^5^	Si1	O2	Al2	−152.9(2)
K1 ^2^	Si2	O5	K1 ^1^	119.62(12)	O6 ^5^	Si1	O2	Al2 ^6^	−10.3(3)
K1 ^1^	Si2	O5	K1 ^2^	−119.62(12)	O6 ^5^	Si1	O3	K1 ^2^	4.85(16)
K1	Si2	O5	K1 ^1^	52.96(14)	O6 ^5^	Si1	O3	Si2	−156.4(3)
K1 ^1^	Si2	O5	Si1 ^1^	142.1(3)	O1 ^3^	Al2	O1	Al2 ^3^	−0.002(0)
K1	Si2	O5	Si1 ^1^	−164.93(16)	O5	Si2	O4	Cr1 ^1^	9.5(4)
K1 ^2^	Si2	O5	Si1 ^1^	−98.3(2)	O5	Si2	O4	Cr1 ^3^	149.8(4)
K1 ^1^	Si2	O3	K1 ^2^	43.71(16)	O5	Si2	O4	Al2 ^1^	9.3(3)
K1	Si2	O3	K1 ^2^	110.44(11)	O5	Si2	O4	Al2 ^3^	152.1(2)
K1 ^1^	Si2	O3	Si1	−155.0(2)	O5	Si2	O6	K1 ^1^	−5.10(14)
K1 ^2^	Si2	O3	Si1	161.3(4)	O5	Si2	O6	K1	114.65(14)
K1	Si2	O3	Si1	−88.3(3)	O5	Si2	O6	Si1 ^4^	−103.8(3)
K1	Si1	O2	Cr1	16.7(5)	O5	Si2	O3	K1 ^2^	−4.67(16)
K1 ^5^	Si1	O2	Cr1 ^6^	45.3(4)	O5	Si2	O3	Si1	156.6(3)
K1 ^2^	Si1	O2	Cr1 ^6^	−73.2(4)	O5 ^7^	Si1	O2	Cr1 ^6^	112.6(4)
K1 ^2^	Si1	O2	Cr1	143.5(4)	O5 ^7^	Si1	O2	Cr1	−30.8(4)
K1 ^5^	Si1	O2	Cr1	−98.1(4)	O5 ^7^	Si1	O2	Al2	−34.5(3)
K1	Si1	O2	Cr1 ^6^	160.0(4)	O5 ^7^	Si1	O2	Al2 ^6^	108.2(3)
K1 ^5^	Si1	O2	Al2	−101.7(2)	O5 ^7^	Si1	O3	K1 ^2^	−110.84(13)
K1 ^2^	Si1	O2	Al2 ^6^	−77.5(3)	O5 ^7^	Si1	O3	Si2	87.9(3)
K1	Si1	O2	Al2^6^	155.7(2)	O3	Si2	O4	Cr1^3^	−91.5(4)
K1	Si1	O2	Al2	13.0(3)	O3	Si2	O4	Cr1 ^1^	128.3(3)
K1 ^2^	Si1	O2	Al2	139.8(2)	O3	Si2	O4	Al2 ^1^	128.0(2)
K1 ^5^	Si1	O2	Al2 ^6^	41.0(3)	O3	Si2	O4	Al2 ^3^	−89.1(2)
K1	Si1	O3	K1 ^2^	−110.63(11)	O3	Si2	O6	K1 ^1^	−120.48(12)
K1 ^5^	Si1	O3	K1 ^2^	−43.80(16)	O3	Si2	O6	K1	−0.72(18)
K1 ^2^	Si1	O3	Si2	−161.2(4)	O3	Si2	O6	Si1 ^4^	140.8(2)
K1	Si1	O3	Si2	88.1(3)	O3	Si2	O5	K1 ^2^	3.97(14)
K1 ^5^	Si1	O3	Si2	155.0(2)	O3	Si2	O5	K1 ^1^	123.59(13)
O2 ^6^	Al2	O1	Al2 ^3^	173.1(3)	O3	Si2	O5	Si1 ^1^	−94.3(2)
O2	Al2	O1	Al2 ^3^	94.0(2)	O3	Si1	O2	Cr1 ^6^	−124.9(4)
O2	Si1	O3	K1 ^2^	125.92(12)	O3	Si1	O2	Cr1	91.8(4)
O2	Si1	O3	Si2	−35.3(3)	O3	Si1	O2	Al2 ^6^	−129.2(3)
O4 ^7^	Al2	O1	Al2 ^3^	−41.1(13)	O3	Si1	O2	Al2	88.1(3)
O4 ^3^	Al2	O1	Al2 ^3^	−93.8(2)					

^1^ 1 + X,+Y,+Z; ^2^ 1/2 + X,1/2 + Y,+Z; ^3^ 1-X,1-Y,1-Z; ^4^ 1/2 + X,-1/2 + Y,+Z; ^5^ -1/2 + X,1/2 + Y,+Z; ^6^ 1/2-X,3/2-Y,1-Z; ^7^ -1 + X,+Y,+Z.

**Table A7:** Fractional atomic coordinates (×10^4^) and equivalent isotropic displacement parameters (Å^2^ × 10^3^) for lepidolite. U_eq_ is defined as 1/3 of the trace of the orthogonalized U_IJ_ tensor.

Atom	*x*	*y*	*z*	U(eq)
Si1	4541(3)	7437(2)	6337.3(9)	15.6(6)
Si2	9635(4)	5734(2)	6336.5(9)	15.2(6)
Al1	2536(5)	9160(2)	4999.5(13)	24.9(7)
O7	4307(12)	9092(5)	6657(3)	25.1(13)
O2	2405(10)	6399(6)	6666(3)	24.5(12)
O3	7414(10)	6827(6)	6588(3)	26.7(13)
O4	3978(10)	7483(5)	5527(2)	20.5(11)
O5	9518(10)	5644(6)	5528(3)	22.1(12)
O6	5476(10)	5670(5)	4510(2)	19.7(11)
Li2	7500	7500	5000	38(5)
Fe1	5000	4060.8(18)	7500	20.3(8)
O1	4850(110)	3850(60)	7010(30)	0(11)

**Table A8:** Anisotropic displacement parameters (Å^2^ × 10^3^) for lepidolite. The Anisotropic displacement factor exponent takes the form: −2π^2^[h^2^a * ^2^U_11_ + 2hka * b * U_12_+…].

Atom	U_11_	U_22_	U_33_	U_23_	U_13_	U_12_
Si1	14.0(10)	14.3(10)	18.0(10)	−0.8(6)	−0.7(7)	−0.6(7)
Si2	15.6(11)	10.2(9)	19.5(11)	−2.0(6)	0.2(8)	−1.0(6)
Al1	24.9(13)	20.3(13)	29.1(14)	−0.5(8)	0.1(10)	−1.2(8)
O7	37(3)	13(3)	25(3)	−2.2(18)	2(2)	0(2)
O2	22(3)	26(3)	25(3)	−1(2)	1(2)	−9(2)
O3	18(3)	33(3)	28(3)	−8(2)	−5(2)	11(2)
O4	22(2)	21(2)	18(3)	−0.8(19)	−1(2)	0(2)
O5	21(3)	23(3)	22(3)	−4(2)	2(2)	−4(2)
O6	26(3)	20(2)	13(2)	1.6(19)	2(2)	−7(2)
Li2	36(11)	43(13)	36(11)	2(9)	7(9)	15(10)
Fe1	18.3(11)	20.2(11)	21.9(13)	0	0.0(7)	0

**Table A9:** Bond lengths for lepidolite.

Atom	Atom	Length/Å	Atom	Atom	Length/Å
Si1	O7	1.636(5)	Al1	O4 ^3^	1.946(5)
Si1	O2	1.641(5)	Al1	O5 ^5^	1.956(6)
Si1	O3	1.628(5)	Al1	O5 ^6^	1.944(6)
Si1	O4	1.632(5)	Al1	O6 ^3^	1.939(6)
Si2	O7 ^1^	1.633(5)	Al1	O6 ^6^	1.943(6)
Si2	O2 ^2^	1.641(5)	Al1	Li2	2.986(2)
Si2	O3	1.637(5)	Al1	Li2 ^6^	3.014(2)
Si2	O5	1.627(5)	Al1	Li2 ^7^	3.019(2)
Al1	Al1 ^3^	2.997(4)	O4	Li2	2.205(5)
Al1	Al1 ^4^	2.979(5)	O5	Li2	2.196(5)
Al1	O4	1.957(5)	O6	Li2	2.148(5)

^1^ 1/2 + X,-1/2 + Y,+Z; ^2^ 1 + X,+Y,+Z; ^3^ 1/2-X,3/2-Y,1-Z; ^4^ 1-X,2-Y,1-Z; ^5^ 3/2-X,3/2-Y,1-Z; ^6^ -1/2 + X,1/2 + Y,+Z; ^7^ -1 + X,+Y,+Z.

**Table A10:** Bond angles for lepidolite.

Atom	Atom	Atom	Angle/˚	Atom	Atom	Atom	Angle/˚
O7	Si1	O2	106.5(3)	Si2	O5	Al1 ^1^	123.4(3)
O3	Si1	O7	106.7(3)	Si2	O5	Al1 ^7^	125.7(3)
O3	Si1	O2	108.6(3)	Si2	O5	Li2	114.7(3)
O3	Si1	O4	113.0(3)	Al1 ^1^	O5	Al1 ^7^	99.6(2)
O4	Si1	O7	111.0(3)	Al1 ^1^	O5	Li2	93.2(2)
O4	Si1	O2	110.8(3)	Al1 ^7^	O5	Li2	91.8(2)
O7 ^1^	Si2	O2 ^2^	107.0(3)	Al1 ^4^	O6	Al1 ^1^	103.3(2)
O7 ^1^	Si2	O3	108.7(3)	Al1 ^4^	O6	Li2	95.1(2)
O3	Si2	O2 ^2^	105.9(3)	Al1 ^1^	O6	Li2	94.8(2)
O5	Si2	O7 ^1^	110.9(3)	Al1 ^7^	Li2	Al1 ^4^	120.13(7)
O5	Si2	O2 ^2^	111.7(3)	Al1 ^7^	Li2	Al1	180.0
O5	Si2	O3	112.4(3)	Al1 ^1^	Li2	Al1 ^4^	60.60(8)
Al1 ^3^	Al1	Al1 ^4^	121.30(16)	Al1 ^7^	Li2	Al1 ^2^	59.87(7)
Al1 ^3^	Al1	Li2	60.70(8)	Al1 ^7^	Li2	Al1 ^3^	120.47(8)
Al1 ^3^	Al1	Li2 ^5^	59.77(7)	Al1 ^4^	Li2	Al1 ^2^	180.00(10)
Al1 ^3^	Al1	Li2 ^6^	179.17(13)	Al1	Li2	Al1 ^2^	120.13(7)
Al1 ^4^	Al1	Li2 ^5^	178.93(14)	Al1	Li2	Al1 ^3^	59.53(8)
Al1 ^4^	Al1	Li2 ^6^	59.53(8)	Al1	Li2	Al1 ^1^	120.47(8)
O4 ^4^	Al1	Al1 ^3^	133.8(2)	Al1 ^3^	Li2	Al1 ^2^	60.60(8)
O4	Al1	Al1 ^3^	96.20(19)	Al1 ^1^	Li2	Al1 ^3^	180.0
O4	Al1	Al1 ^4^	39.70(15)	Al1 ^7^	Li2	Al1 ^1^	59.53(8)
O4 ^4^	Al1	Al1 ^4^	39.98(15)	Al1 ^3^	Li2	Al1 ^4^	119.40(8)
O4 ^4^	Al1	O4	79.7(2)	Al1	Li2	Al1 ^4^	59.87(7)
O4 ^4^	Al1	O5 ^7^	94.0(2)	Al1 ^1^	Li2	Al1 ^2^	119.40(8)
O4 ^4^	Al1	Li2 ^6^	46.83(16)	O4	Li2	Al1 ^7^	139.07(14)
O4	Al1	Li2	47.57(16)	O4	Li2	Al1 ^1^	89.93(14)
O4 ^4^	Al1	Li2	85.56(17)	O4 ^7^	Li2	Al1 ^3^	89.93(14)
O4	Al1	Li2 ^5^	140.81(18)	O4	Li2	Al1 ^3^	90.07(14)
O4	Al1	Li2 ^6^	84.47(16)	O4 ^7^	Li2	Al1 ^7^	40.93(14)
O4 ^4^	Al1	Li2 ^5^	139.49(18)	O4	Li2	Al1 ^2^	139.94(14)
O5 ^5^	Al1	Al1 ^4^	134.0(2)	O4^7^	Li2	Al1 ^2^	40.06(14)
O5 ^7^	Al1	Al1 ^4^	95.80(19)	O4	Li2	Al1 ^4^	40.06(14)
O5 ^7^	Al1	Al1 ^3^	40.06(16)	O4^7^	Li2	Al1	139.07(14)
O5 ^5^	Al1	Al1 ^3^	40.35(16)	O4	Li2	Al1	40.93(14)
O5 ^7^	Al1	O4	94.9(2)	O4 ^7^	Li2	Al1 ^4^	139.94(14)
O5 ^5^	Al1	O4	94.6(3)	O4 ^7^	Li2	Al1 ^1^	90.07(14)
O5 ^5^	Al1	O4 ^4^	171.7(3)	O4 ^7^	Li2	O4	180.0
O5 ^5^	Al1	O5 ^7^	80.4(2)	O5	Li2	Al1 ^3^	139.91(14)
O5 ^5^	Al1	Li2 ^6^	139.15(19)	O5 ^7^	Li2	Al1 ^2^	89.76(14)
O5 ^5^	Al1	Li2	86.12(18)	O5 ^7^	Li2	Al1 ^7^	139.10(14)
O5 ^7^	Al1	Li2 ^6^	140.44(18)	O5 ^7^	Li2	Al1	40.90(14)
O5 ^7^	Al1	Li2 ^5^	85.13(17)	O5 ^7^	Li2	Al1 ^4^	90.24(14)
O5 ^7^	Al1	Li2	47.32(17)	O5	Li2	Al1 ^1^	40.09(14)
O5 ^5^	Al1	Li2 ^5^	46.68(17)	O5	Li2	Al1 ^4^	89.76(14)
O6 ^5^	Al1	Al1 ^3^	94.37(19)	O5	Li2	Al1	139.10(14)
O6 ^4^	Al1	Al1 ^3^	134.2(2)	O5	Li2	Al1 ^7^	40.90(14)
O6 ^4^	Al1	Al1 ^4^	93.91(19)	O5	Li2	Al1 ^2^	90.24(14)
O6 ^5^	Al1	Al1 ^4^	134.0(2)	O5 ^7^	Li2	Al1 ^1^	139.91(14)
O6 ^4^	Al1	O4 ^4^	91.9(2)	O5 ^7^	Li2	Al1 ^3^	40.09(14)
O6 ^4^	Al1	O4	94.0(2)	O5 ^7^	Li2	O4	81.83(19)
O6 ^5^	Al1	O4 ^4^	94.7(2)	O5 ^7^	Li2	O4 ^7^	98.17(19)
O6 ^5^	Al1	O4	169.2(3)	O5	Li2	O4	98.17(19)
O6 ^5^	Al1	O5 ^7^	94.8(2)	O5	Li2	O4 ^7^	81.83(19)
O6 ^4^	Al1	O5 ^5^	94.5(2)	O5	Li2	O5 ^7^	180.0
O6 ^5^	Al1	O5 ^5^	91.9(2)	O6	Li2	Al1 ^3^	140.03(15)
O6 ^4^	Al1	O5 ^7^	170.0(2)	O6	Li2	Al1 ^4^	39.77(14)
O6 ^4^	Al1	O6 ^5^	76.7(2)	O6 ^7^	Li2	Al1 ^3^	39.97(15)
O6 ^5^	Al1	Li2 ^5^	45.25(16)	O6	Li2	Al1 ^7^	90.00(15)
O6 ^4^	Al1	Li2 ^6^	45.14(15)	O6 ^7^	Li2	Al1 ^2^	39.77(14)
O6 ^5^	Al1	Li2 ^6^	84.95(17)	O6 ^7^	Li2	Al1 ^4^	140.23(14)
O6 ^4^	Al1	Li2 ^5^	85.14(16)	O6	Li2	Al1	90.00(15)
O6 ^4^	Al1	Li2	141.39(18)	O6	Li2	Al1 ^2^	140.23(14)
O6 ^5^	Al1	Li2	141.87(19)	O6 ^7^	Li2	Al1	90.00(15)
Li2	Al1	Al1 ^4^	60.60(8)	O6 ^7^	Li2	Al1 ^1^	140.03(15)
Li2	Al1	Li2 ^6^	120.13(7)	O6	Li2	Al1 ^1^	39.97(15)
Li2 ^5^	Al1	Li2 ^6^	119.40(8)	O6 ^7^	Li2	Al1 ^7^	90.00(15)
Li2	Al1	Li2 ^5^	120.47(8)	O6 ^7^	Li2	O4 ^7^	79.81(19)
Si2 ^5^	O7	Si1	131.2(4)	O6 ^7^	Li2	O4	100.19(18)
Si1	O2	Si2 ^6^	130.4(3)	O6	Li2	O4	79.81(19)
Si1	O3	Si2	139.7(3)	O6	Li2	O4 ^7^	100.19(18)
Si1	O4	Al1	125.9(3)	O6 ^7^	Li2	O5 ^7^	80.03(19)
Si1	O4	Al1^4^	123.4(3)	O6	Li2	O5	80.03(19)
Si1	O4	Li2	113.9(3)	O6 ^7^	Li2	O5	99.97(19)
Al1 ^4^	O4	Al1	100.3(2)	O6	Li2	O5 ^7^	99.97(19)
Al1 ^4^	O4	Li2	93.1(2)	O6 ^7^	Li2	O6	180.0
Al1	O4	Li2	91.5(2)				

^1^ 1/2 + X,-1/2+Y,+Z; ^2^ 1 + X,+Y,+Z; ^3^ 1-X,2-Y,1-Z; ^4^ 1/2-X,3/2-Y,1-Z; ^5^ -1/2 + X,1/2 + Y,+Z; ^6^ -1 + X,+Y,+Z; ^7^ 3/2-X,3/2-Y,1-Z.

**Table A11:** Torsion angles for lepidolite.

A	B	C	D	Angle/˚	A	B	C	D	Angle/˚
O7	Si1	O2	Si2 ^1^	107.0(5)	O2 ^6^	Si2	O5	Al1 ^4^	13.5(5)
O7	Si1	O3	Si2	−149.6(6)	O2 ^6^	Si2	O5	Al1 ^3^	149.5(3)
O7	Si1	O4	Al1 ^2^	−149.6(4)	O2 ^6^	Si2	O5	Li2	−98.4(3)
O7	Si1	O4	Al1	−11.7(5)	O3	Si1	O7	Si2 ^5^	99.0(5)
O7	Si1	O4	Li2	99.1(3)	O3	Si1	O2	Si2 ^1^	−138.5(4)
O7 ^3^	Si2	O3	Si1	−95.5(6)	O3	Si1	O4	Al1 ^2^	90.6(4)
O7 ^3^	Si2	O5	Al1 ^3^	30.2(5)	O3	Si1	O4	Al1	−131.5(4)
O7 ^3^	Si2	O5	Al1 ^4^	−105.8(4)	O3	Si1	O4	Li2	−20.7(4)
O7 ^3^	Si2	O5	Li2	142.3(3)	O3	Si2	O5	Al1 ^3^	−91.6(4)
O2	Si1	O7	Si2 ^5^	−145.1(5)	O3	Si2	O5	Al1 ^4^	132.3(4)
O2	Si1	O3	Si2	96.0(6)	O3	Si2	O5	Li2	20.5(4)
O2	Si1	O4	Al1	106.3(4)	O4	Si1	O7	Si2 ^5^	−24.5(6)
O2	Si1	O4	Al1 ^2^	−31.5(4)	O4	Si1	O2	Si2 ^1^	−13.8(5)
O2	Si1	O4	Li2	−142.8(3)	O4	Si1	O3	Si2	−27.4(7)
O2 ^6^	Si2	O3	Si1	149.9(6)	O5	Si2	O3	Si1	27.6(7)

^1^ -1 + X,+Y,+Z; ^2^ 1/2-X,3/2-Y,1-Z; ^3^ 1/2 + X,-1/2 + Y,+Z; ^4^ 3/2-X,3/2-Y,1-Z; ^5^ -1/2 + X,1/2 + Y,+Z; ^6^ 1 + X,+Y,+Z.

**Table A12:** Fractional Atomic Coordinates (×10^4^) and Equivalent Isotropic Displacement Parameters (Å^2^ × 10^3^) for Spodumene. U_eq_ is defined as 1/3 of the trace of the orthogonalized U_IJ_ tensor.

Atom	*x*	*y*	*z*	U(eq)
Si1	4619.7(9)	4066.4(5)	2058.8(5)	6.4(3)
Al1	2500	933.4(8)	0	6.2(3)
O1	5308(2)	4178.0(13)	3901.6(13)	7.6(3)
O3	4359(2)	2331.2(14)	1352.0(13)	10.6(3)
O2	2022(2)	5130.5(14)	1434.8(12)	10.3(3)
Li1	7500	2265(6)	5000	17.0(9)

**Table A13:** Anisotropic displacement parameters (Å^2^ × 10^3^) for spodumene. The anisotropic displacement factor exponent takes the form: −2π^2^[h^2^a * ^2^U_11_ + 2hka * b * U_12_+…].

Atom	U_11_	U_22_	U_33_	U_23_	U_13_	U_12_
Si1	6.6(4)	6.9(4)	5.8(3)	−0.67(14)	1.2(2)	−0.20(15)
Al1	7.1(5)	6.0(4)	5.5(4)	0	1.3(3)	0
O1	8.6(6)	8.1(7)	6.3(6)	−0.5(4)	2.1(5)	−0.2(4)
O3	12.6(7)	8.5(7)	10.3(6)	−2.3(4)	1.5(5)	0.1(5)
O2	10.1(7)	12.7(6)	8.0(7)	0.6(4)	1.8(5)	2.7(5)
Li1	19(2)	17(2)	15(2)	0	3.9(18)	0

**Table A14:** Bond lengths for Spodumene.

Atom	Atom	Length/Å	Atom	Atom	Length/Å
Si1	O1	1.6404(12)	Al1	O1 ^6^	1.9459(12)
Si1	O3	1.5863(12)	Al1	O3 ^7^	1.8219(12)
Si1	O2^1^	1.6294(12)	Al1	O3	1.8219(12)
Si1	O2	1.6215(12)	Al1	Li1 ^5^	3.079(5)
Si1	Li1^2^	2.8688(19)	Al1	Li1 ^3^	3.017(2)
Si1	Li1	3.159(2)	Al1	Li1 ^2^	3.017(2)
Al1	O1 ^3^	1.9459(12)	O1	Li1	2.096(4)
Al1	O1 ^4^	1.9970(12)	O3	Li1 ^2^	2.2767(14)
Al1	O1 ^5^	1.9970(12)	O2	Li1 ^8^	2.262(4)

^1^ 1/2 + X,1-Y,+Z; ^2^ 3/2-X,1/2-Y,1/2-Z; ^3^ 1/2-X,1/2-Y,1/2-Z; ^4^ 1-X,-1/2 + Y,1/2-Z; ^5^ -1/2 + X,-1/2 + Y,-1/2 + Z; ^6^ +X,1/2-Y,-1/2 + Z; ^7^ 1/2-X,+Y,-Z; ^8^ -1/2 + X,1/2 + Y,-1/2 + Z.

**Table A15:** Bond angles for spodumene.

Atom	Atom	Atom	Angle/˚	Atom	Atom	Atom	Angle/˚
O1	Si1	Li1	37.00(8)	Si1 ^1^	Li1	Si1	81.615(18)
O1	Si1	Li1 ^1^	131.24(6)	Si1 ^1^	Li1	Al1 ^1^	67.695(17)
O3	Si1	O1	116.60(6)	Si1 ^11^	Li1	Al1 ^1^	140.37(3)
O3	Si1	O2 ^2^	104.13(7)	Si1 ^1^	Li1	Al1 ^8^	112.94(9)
O3	Si1	O2	111.80(7)	Si1 ^11^	Li1	Al1 ^6^	67.695(17)
O3	Si1	Li1 ^1^	52.32(9)	Si1 ^11^	Li1	Al1 ^8^	112.94(9)
O3	Si1	Li1	83.56(9)	Si1 ^1^	Li1	Al1 ^6^	140.37(3)
O2	Si1	O1	108.04(6)	Al1 ^1^	Li1	Si1	90.84(9)
O2 ^2^	Si1	O1	108.45(6)	Al1 ^8^	Li1	Si1	61.41(7)
O2	Si1	O2 ^2^	107.36(4)	Al1 ^6^	Li1	Si1	60.38(5)
O2 ^2^	Si1	Li1 ^1^	51.92(9)	Al1 ^1^	Li1	Al1 ^8^	59.92(8)
O2	Si1	Li1 ^1^	120.16(6)	Al1 ^1^	Li1	Al1 ^6^	119.83(15)
O2	Si1	Li1	140.39(5)	Al1 ^6^	Li1	Al1 ^8^	59.92(8)
O2 ^2^	Si1	Li1	103.38(5)	O1	Li1	Si1	28.10(3)
Li1 ^1^	Si1	Li1	98.386(18)	O1 ^12^	Li1	Si1	98.58(15)
O1 ^3^	Al1	O1 ^4^	96.97(5)	O1	Li1	Si1 ^1^	107.22(5)
O1 ^4^	Al1	O1 ^5^	84.90(7)	O1	Li1	Si1 ^11^	107.51(6)
O1 ^6^	Al1	O1 ^5^	96.97(5)	O1 ^12^	Li1	Si1 ^1^	107.51(6)
O1 ^6^	Al1	O1 ^4^	78.91(5)	O1 ^12^	Li1	Si1 ^11^	107.22(5)
O1 ^3^	Al1	O1 ^5^	78.91(5)	O1	Li1	Al1 ^8^	40.01(9)
O1 ^3^	Al1	O1 ^6^	174.49(7)	O1 ^12^	Li1	Al1 ^1^	39.86(5)
O1 ^3^	Al1	Li1 ^1^	43.67(7)	O1 ^12^	Li1	Al1 ^6^	89.99(13)
O1 ^4^	Al1	Li1 ^4^	42.45(3)	O1	Li1	Al1 ^1^	89.99(13)
O1 ^6^	Al1	Li1 ^1^	140.49(6)	O1 ^12^	Li1	Al1 ^8^	40.01(9)
O1 ^3^	Al1	Li1 ^6^	140.49(6)	O1	Li1	Al1 ^6^	39.86(5)
O1 ^4^	Al1	Li1 ^6^	88.12(7)	O1	Li1	O1 ^12^	80.03(18)
O1 ^5^	Al1	Li1 ^1^	88.12(7)	O1 ^12^	Li1	O3 ^1^	76.18(10)
O1 ^3^	Al1	Li1 ^4^	87.25(4)	O1	Li1	O3 ^11^	76.18(10)
O1 ^6^	Al1	Li1 ^4^	87.25(4)	O1 ^12^	Li1	O3 ^11^	90.64(12)
O1 ^6^	Al1	Li1 ^6^	43.67(7)	O1	Li1	O3 ^1^	90.64(12)
O1 ^4^	Al1	Li1 ^1^	140.59(6)	O1 ^12^	Li1	O2 ^13^	116.63(4)
O1 ^5^	Al1	Li1 ^6^	140.59(6)	O1	Li1	O2 ^13^	139.93(5)
O1 ^5^	Al1	Li1 ^4^	42.45(3)	O1 ^12^	Li1	O2 ^5^	139.93(5)
O3 ^7^	Al1	O1 ^4^	88.42(5)	O1	Li1	O2 ^5^	116.63(4)
O3	Al1	O1 ^4^	167.96(5)	O3 ^1^	Li1	Si1 ^1^	33.47(4)
O3	Al1	O1 ^3^	91.54(5)	O3 ^11^	Li1	Si1 ^1^	161.83(16)
O3 ^7^	Al1	O1 ^3^	92.00(5)	O3 ^11^	Li1	Si1 ^11^	33.47(4)
O3	Al1	O1 ^5^	88.42(5)	O3 ^1^	Li1	Si1	74.82(6)
O3 ^7^	Al1	O1 ^6^	91.54(5)	O3 ^11^	Li1	Si1	96.87(8)
O3 ^7^	Al1	O1 ^5^	167.96(5)	O3 ^1^	Li1	Si1 ^11^	161.83(16)
O3	Al1	O1 ^6^	92.00(5)	O3 ^11^	Li1	Al1 ^6^	37.04(5)
O3	Al1	O3 ^7^	99.81(8)	O3 ^1^	Li1	Al1 ^8^	81.45(12)
O3	Al1	Li1 ^4^	130.10(4)	O3 ^11^	Li1	Al1 ^1^	130.47(15)
O3 ^7^	Al1	Li1 ^6^	48.83(5)	O3 ^1^	Li1	Al1 ^1^	37.04(5)
O3	Al1	Li1 ^1^	48.83(5)	O3 ^11^	Li1	Al1 ^8^	81.45(12)
O3 ^7^	Al1	Li1 ^4^	130.10(4)	O3 ^1^	Li1	Al1^6^	130.47(15)
O3 ^7^	Al1	Li1 ^1^	90.72(7)	O3 ^1^	Li1	O3 ^11^	162.9(2)
O3	Al1	Li1 ^6^	90.72(7)	O2 ^13^	Li1	Si1 ^1^	101.91(15)
Li1 ^1^	Al1	Li1 ^6^	119.83(15)	O2 ^13^	Li1	Si1	140.94(9)
Li1 ^6^	Al1	Li1 ^4^	120.08(8)	O2 ^5^	Li1	Si1	88.95(3)
Li1 ^1^	Al1	Li1 ^4^	120.08(8)	O2 ^5^	Li1	Si1 ^1^	34.53(4)
Si1	O1	Al1 ^6^	119.89(7)	O2 ^5^	Li1	Si1 ^11^	101.91(15)
Si1	O1	Al1 ^8^	122.01(7)	O2 ^13^	Li1	Si1 ^11^	34.53(4)
Si1	O1	Li1	114.90(8)	O2 ^13^	Li1	Al1 ^6^	101.20(4)
Al1 ^6^	O1	Al1 ^8^	101.09(5)	O2 ^5^	Li1	Al1 ^6^	126.86(6)
Al1 ^8^	O1	Li1	97.54(10)	O2 ^5^	Li1	Al1 ^8^	142.39(8)
Al1 ^6^	O1	Li1	96.47(6)	O2 ^13^	Li1	Al1 ^8^	142.39(8)
Si1	O3	Al1	148.44(8)	O2 ^13^	Li1	Al1 ^1^	126.86(6)
Si1	O3	Li1 ^1^	94.21(13)	O2 ^5^	Li1	Al1 ^1^	101.20(4)
Al1	O3	Li1 ^1^	94.13(9)	O2 ^13^	Li1	O3 ^1^	127.67(16)
Si1	O2	Si1 ^9^	138.97(8)	O2 ^5^	Li1	O3 ^11^	127.67(16)
Si1 ^9^	O2	Li1 ^10^	93.55(8)	O2 ^13^	Li1	O3 ^11^	67.94(7)
Si1	O2	Li1 ^10^	116.79(7)	O2 ^5^	Li1	O3 ^1^	67.94(7)
Si1 ^1^	Li1	Si1 ^11^	134.13(17)	O2 ^5^	Li1	O2 ^13^	75.23(16)
Si1 ^11^	Li1	Si1	121.25(2)				

^1^ 3/2-X,1/2-Y,1/2-Z; ^2^ 1/2 + X,1-Y,+Z; ^3^ +X,1/2-Y,-1/2 + Z; ^4^ -1/2 + X,-1/2+Y,-1/2 + Z; ^5^ 1-X,-1/2 + Y,1/2-Z; ^6^ 1/2-X,1/2-Y,1/2-Z; ^7^ 1/2-X,+Y,-Z; ^8^ ½ + X,1/2 + Y,1/2+Z; ^9^ -1/2 + X,1-Y,+Z; ^10^ -1/2 + X,1/2 + Y,-1/2 + Z; ^11^ +X,1/2-Y,1/2 + Z; ^12^ 3/2-X,+Y,1-Z; ^13^ 1/2 + X,-1/2 + Y,1/2 + Z.

**Table A16:** Torsion angles for spodumene.

A	B	C	D	Angle/˚	A	B	C	D	Angle/˚
O1	Si1	O3	Al1	132.06(14)	O2	Si1	O1	Li1	156.07(9)
O1	Si1	O3	Li1 ^1^	−123.01(7)	O2	Si1	O3	Al1	7.07(17)
O1	Si1	O2	Si1 ^2^	−16.87(14)	O2 ^10^	Si1	O3	Al1	−108.53(15)
O1	Si1	O2	Li1 ^3^	116.44(10)	O2 ^10^	Si1	O3	Li1 ^1^	−3.60(7)
O1 ^4^	Al1	O3	Si1	114.97(15)	O2	Si1	O3	Li1 ^1^	112.00(7)
O1 ^5^	Al1	O3	Si1	−166.17(15)	O2 ^10^	Si1	O2	Si1 ^2^	−133.64(13)
O1 ^6^	Al1	O3	Si1	−69.24(15)	O2 ^10^	Si1	O2	Li1 ^3^	−0.34(12)
O1 ^7^	Al1	O3	Si1	−109.9(3)	Li1 ^1^	Si1	O1	Al1 ^6^	−146.96(11)
O1 ^4^	Al1	O3	Li1^1^	10.01(11)	Li1 ^1^	Si1	O1	Al1 ^8^	84.66(14)
O1 ^6^	Al1	O3	Li1 ^1^	−174.20(10)	Li1	Si1	O1	Al1 ^6^	−114.20(12)
O1 ^7^	Al1	O3	Li1 ^1^	145.1(3)	Li1	Si1	O1	Al1 ^8^	117.42(12)
O1 ^5^	Al1	O3	Li1 ^1^	88.88(10)	Li1 ^1^	Si1	O1	Li1	−32.8(2)
O3	Si1	O1	Al1 ^8^	146.63(7)	Li1	Si1	O3	Al1	149.25(15)
O3	Si1	O1	Al1 ^6^	−85.00(9)	Li1 ^1^	Si1	O3	Al1	−104.93(16)
O3	Si1	O1	Li1	29.20(12)	Li1	Si1	O3	Li1^1^	−105.82(6)
O3	Si1	O2	Si1 ^2^	112.74(12)	Li1 ^1^	Si1	O2	Si1 ^2^	170.80(13)
O3	Si1	O2	Li1 ^3^	−113.96(10)	Li1	Si1	O2	Si1 ^2^	5.6(2)
O3 ^9^	Al1	O3	Si1	22.67(12)	Li1	Si1	O2	Li1 ^3^	138.95(19)
O3 ^9^	Al1	O3	Li1 ^1^	−82.29(10)	Li1 ^1^	Si1	O2	Li1 ^3^	−55.89(18)
O2	Si1	O1	Al1 ^8^	−86.50(9)	Li1 ^1^	Al1	O3	Si1	104.96(18)
O2 ^10^	Si1	O1	Al1 ^6^	157.94(7)	Li1 ^7^	Al1	O3	Si1	−157.33(12)
O2	Si1	O1	Al1 ^6^	41.87(9)	Li1 ^6^	Al1	O3	Si1	−25.58(15)
O2 ^10^	Si1	O1	Al1 ^8^	29.57(9)	Li1 ^7^	Al1	O3	Li1 ^1^	97.71(10)
O2 ^10^	Si1	O1	Li1	−87.86(10)	Li1 ^6^	Al1	O3	Li1 ^1^	−130.53(14)

^1^ 3/2-X,1/2-Y,1/2-Z; ^2^ -1/2 + X,1-Y,+Z; ^3^ -1/2 + X,1/2 + Y,-1/2 + Z; ^4^ +X,1/2-Y,-1/2 + Z; ^5^ 1-X,-1/2 + Y,1/2-Z; ^6^ 1/2-X,1/2-Y,1/2-Z; ^7^ -1/2 + X,-1/2 + Y,-1/2 + Z; ^8^ 1/2 + X,1/2 + Y,1/2 + Z; ^9^ 1/2-X,+Y,-Z; ^10^ 1/2 + X,1-Y,+Z.
